# Laser‐Induced Covalent Defunctionalization of Graphene — Precise Patterning and Site‐Selective Removal of Functional Groups

**DOI:** 10.1002/advs.202511481

**Published:** 2025-08-30

**Authors:** Tamara Nagel, Kevin Gerein, Frank Hauke, Andreas Hirsch

**Affiliations:** ^1^ Department of Chemistry and Pharmacy & Center of Advanced Materials and Processes (ZMP) Friedrich‐Alexander Universität Erlangen‐Nürnberg Nikolaus‐Fiebiger‐Str. 10 91058 Erlangen Germany

**Keywords:** graphene, molecular information storage, nanoscale patterning, Raman spectroscopy, site‐selective defunctionalization

## Abstract

In this study, for the first time, site‐selective defunctionalization concepts for structuring and reusing covalently patterned monolayer graphene are reported. Using a laser‐activated precursor deposition approach with dibenzoyl peroxide (DBPO), phenyl moieties are covalently grafted onto graphene with high spatial precision. Temperature‐dependent Raman spectroscopy reveals that functionalization is fully reversible, with lattice‐scale defunctionalization occurring at 225 °C, independent of the initial functionalization degree. By applying high‐power laser irradiation (*λ_exc_*_._ = 532 nm) the covalent addends can be selectively removed with high local control, as confirmed by Raman mapping and Kelvin probe force microscopy (KPFM). This photothermal process enables a lateral defunctionalization resolution of ≈0.5 µm. Importantly, it is demonstrated that “erased” regions can be successfully refunctionalized using a second laser “writing” sequence, achieving highly reproducible functionalization levels. The ability to iteratively “write,” “erase,” and “rewrite” covalent functionalities with a precise control of the grafting pattern establishes graphene as a promising platform for chemically tunable, high‐resolution 2D data storage.

## Graphical abstract

Laser‐triggered graphene defunctionalization: Study demonstrates controlled removal of functional groups caused by thermal heating or laser irradiation, facilitating local control. Laser‐induced refunctionalization highlights graphene's potential as an information storage medium.

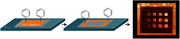

## 1 Introduction

The local structuring of two‐dimensional (2D) materials, particularly graphene, is of great interest across various fields ranging from chemistry and physics to materials science and nanoelectronics. This spatially controlled tailoring of graphene's extraordinary properties enables the introduction of distinct chemical and physical characteristics in adjacent domains. In recent years, several techniques for spatially controlled functionalization of graphene have been developed.^[^ ^1^, ^2^ ^]^ These can be categorized into two main approaches toward 2D structuring of covalently bound substituents on graphene. The first strategy restricts the chemical reaction of the material to pre‐selected regions by masking sites, where covalent attachment is to be prevented,^[^ ^3^, ^4^, ^5^, ^6^ ^]^ resulting in site‐selective property changes in the material. The second approach uses locally controlled activation stimuli, such as laser irradiation^[^ ^7^ ^]^ or plasma exposure,^[^ ^8^ ^]^ to induce the chemical reaction in defined areas. In this way, the activation source can be moved across the graphene surface with almost no restrictions on the pattern shape and trigger the chemical functionalization specifically in the irradiated areas.^[^ ^7^, ^9^, ^10^, ^11^, ^12^, ^13^ ^]^ So far, the removal of functional groups from functionalized graphene has been limited to thermal annealing^[^ ^14^, ^15^ ^]^ or electrochemical processes.^[^ ^16^ ^]^ These methods, however, preclude local control in the defunctionalization process, since both exert forces uniformly over the entire graphene lattice.

Detailed investigations into the thermal defunctionalization of bulk graphene revealed that the varying binding sites of functional moieties on small flakes (rim, edge near positions, in‐plane position) result in a continuous defunctionalization process across a broad temperature range, with defects persisting in the graphene lattice even above 500 °C.^[^ ^17^ ^]^ In contrast, larger graphene sheets exhibit nearly complete defunctionalization, suggesting full reversibility of covalent functionalization in in‐plane positions.^[^ ^15^, ^18^ ^]^

An innovative approach, in contrast to the previously discussed “functional patterning” of graphene, would be to reverse the patterning process by selectively removing functional groups from the functionalized graphene, thereby generating a “restored graphene pattern.”

Recently, initial evidence for laser‐induced local defunctionalization has been demonstrated by our group using suspended graphene^[^ ^19^ ^]^ in analogy to the well‐established concept of “laser‐triggered reduction of graphene oxide (GO).”^[^ ^20^, ^21^, ^22^ ^]^ These promising results may pave the way for a potential application of graphene functionalization as an information storage system, where “functional patterning” generates defined information that could be “erased” by a temperature‐driven or laser‐initiated process.

In this work, we present a pioneering investigation of the thermal defunctionalization of graphene functionalized by laser “writing” with dibenzoyl peroxide (DBPO) based on an in situ scanning Raman microscopy (SRM) monitoring at an unprecedented level. For the first time, the temperature “erased” pristine graphene has been reused for a subsequent covalent functionalization step. Additionally, we have extended this concept to site‐selective defunctionalization in individually targeted areas in a controlled and patterned manner. This novel approach is based on spatially resolved high‐power laser irradiation of covalently functionalized graphene on a substrate and offers a significant flexibility in controlling the shape, area, and lateral dimensions of the resulting “inverse patterns”, which are further analyzed in this work. Together with our in‐depth study of the laser “rewriting” on the previously laser “erased” areas we can now provide a complete cycle of spatially controlled functionalization (“writing”), site‐selective defunctionalization (“erasing”) and a second site‐selective functionalization step (“rewriting”), representing an important step forward towards reusable 2D chemical information storage devices.

## 2 Results and Discussion

**Scheme 1:**
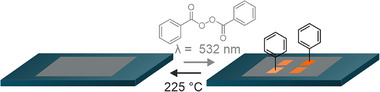
Schematic of laser‐induced phenyl group functionalization of monolayer graphene on a Si/SiO_2_ substrate and the subsequent reversible temperature‐induced defunctionalization.

Reaching the challenging goal of investigating the temperature‐induced detachment of functional moieties bound to graphene in detail has not been possible in the past due to the polydispersity of the functionalized material. Based on the high control of the functional group grafting on monolayer graphene by laser‐triggered activation of suitable precursors,^[^ ^10^, ^11^, ^12^ ^]^ we are now in a position to study the temperature‐driven defunctionalization in detail. In a first step, monolayer graphene deposited on a Si/SiO_2_ substrate was functionalized by applying our optimized laser “writing” approach with DBPO.^[^ ^10^ ^]^ Using this technique, we were able to attach phenyl moieties covalently bound to graphene with high spatial control and fine‐tune the degree of functionalization by a precise adjustment of the laser “writing” parameters. In this way, a sample with four regions with alternating degrees of functionalization was created (**Scheme** **1**).

For a deeper understanding of the thermally induced graphene defunctionalization process, we performed temperature‐dependent scanning Raman microscopy (SRM). For this purpose, the phenyl‐functionalized graphene on a Si/SiO_2_ substrate was placed in a Linkam heating stage and heated stepwise under N_2_ flow. To ensure full thermal equilibrium conditions at each temperature, the corresponding Raman mapping was performed 10 min after reaching the set temperature.

**Figure 1:**
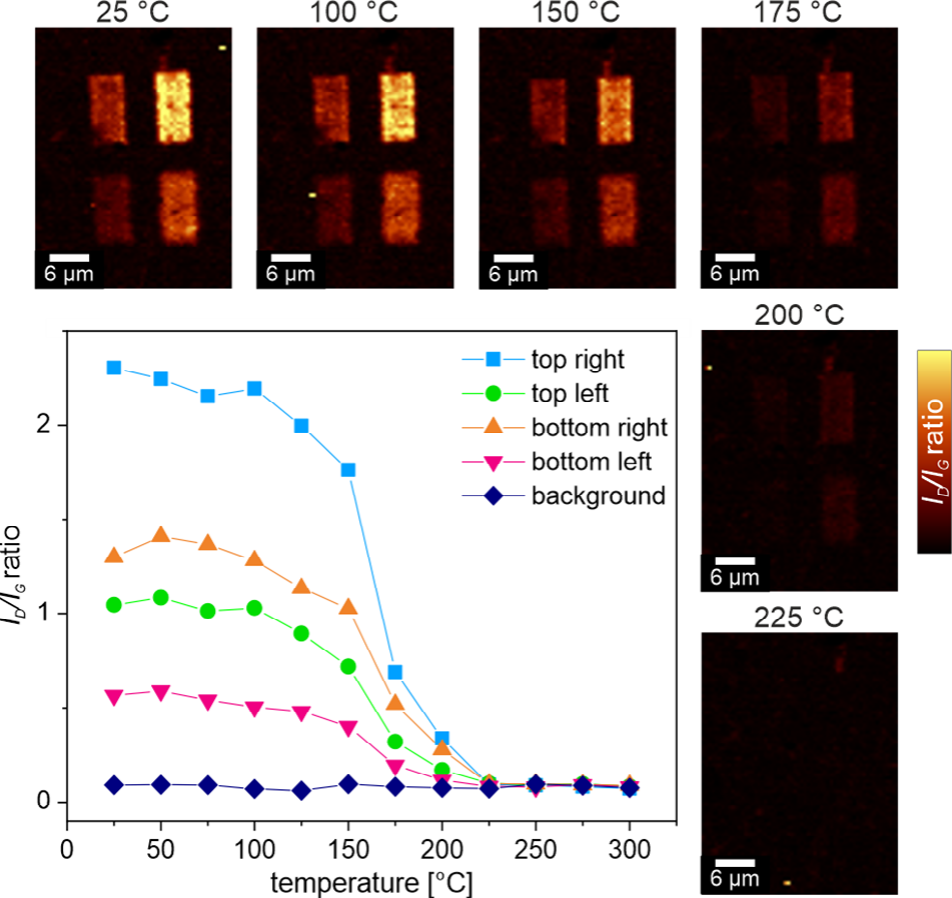
Raman spectroscopic monitoring of the thermally induced defunctionalization of DBPO functionalized graphene: *I*_D_ */I*_G_ “readout” Raman mappings of graphene showing four areas with altering degrees of functionalization (indicated by lighter color for higher addend density) at selected temperatures, (see Figure S1, for further details, Supporting Information) and the respective evolution of the *I*_D_/*I*_G_ ratios as a function of temperature. Each rectangular region represents covalently functionalized graphene with different degrees of functionalization.

**Figure** **1** shows the corresponding *I*_D_/*I*_G_ Raman mappings at distinct sample temperatures. As indicated by the color gradient, the maps represent the change in concentration of covalently attached functional moieties during the temperature‐driven defunctionalization process. Pristine graphene exhibits a prominent *G* band at ≈1582 cm^−1^ and an intense 2D band at ≈2680 cm^−1^, both indicative of an intact, defect‐free sp^2^ lattice. Upon covalent functionalization, lattice sp^3^‐hybridized carbon atoms are generated, resulting in an activation of the Raman *D* band at ≈1350 cm^−1^ (for the evolution of the characteristic 2D band see Figure S1, Supporting Information, presentation of the individual spectra in Figure S2, Supporting Information). The different colors of the four functionalized regions at 25 °C in Figure 1 confirm the different degrees of functionalization in the starting material.

At ≈100 °C, the *I*_D_/*I*_G_ ratio of all four regions begins to decrease (Figure 1), indicating a temperature‐induced detachment of the bound phenyl substituents. Regardless of the initial degree of covalent grafting, all regions exhibit a rather coherent behavior upon thermal heating, with a significant decrease in the *I*_D_ */I*_G_ ratio starting at 150 °C. At a temperature of 225 °C, they all exhibit the Raman properties of defect‐free graphene and become indistinguishable from the unfunctionalized background. These results strongly emphasize the complete reversibility of covalent functional group binding to graphene. The temperature‐induced defunctionalization is independent of the initial degree of functionalization, and the entire chemical information stored in covalently functionalized graphene can be completely erased by heating the sample to 225 °C, which can be compared to the “format” command used for deleting all stored information on a hard disk.

Until now, the “rewritability” of graphene after selective or unselective information “erasure” was unexplored, representing an essential feature for reusable devices. Our current study demonstrates for the first time that thermally erased graphene can be effectively reused in a second laser‐triggered “writing” sequence, marking a significant advancement towards practical implementation.

**Figure 2:**
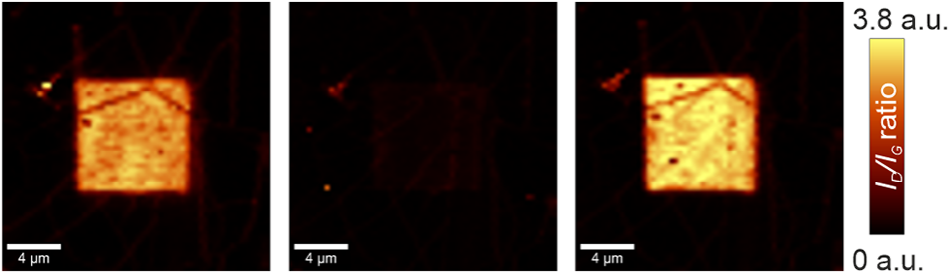
*I*_D_/*I*_G_ map of covalently functionalized graphene with DBPO (left), the same area after temperature‐induced defunctionalization performed at 255 °C (center), and reattached phenyl substituents implemented in a second laser‐triggered functionalization step applying the same laser “writing” parameters as for the first “writing” (right) (see Figure S3, Supporting Information, for further details).

For this purpose, a thermally “erased” monolayer graphene sample was coated with DBPO by spin coating and then irradiated with a green laser (λ_exc_. = 532 nm). As can clearly be seen in **Figure** **2**, this again resulted in a highly efficient, laser path‐selective grafting of phenyl substituents onto the thermally regenerated graphene surface, proving that graphene can be successfully used in iterative “writing”/“erasing” cycles (see Figure S3, for more information, Supporting Information).

**Figure 3:**
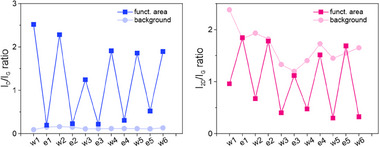
Evolution of the *I*_D_/*I*_G_ and *I*_2D_/*I*_G_ ratio in a multi‐step procedure of thermal “erasing” and laser‐induced “rewriting” with DBPO (see Figures S4–S6, Supporting Information, for further details).

Building on this, we conducted multiple “erasing” and “rewriting” cycles. Each step demonstrated successful defunctionalization and subsequent refunctionalization, thereby confirming the platform's durability for at least five cycles (**Figure** **3**).

**Scheme 2:**
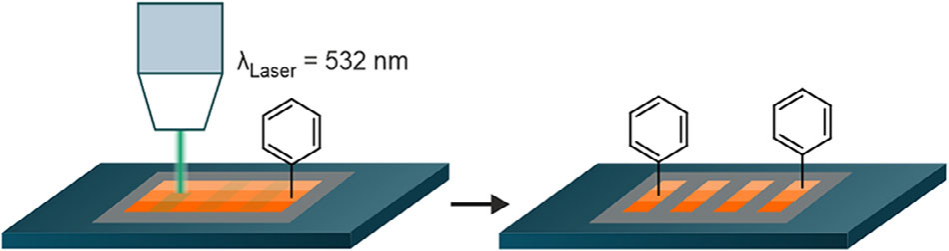
Schematic of the site‐specific laser‐triggered defunctionalization of graphene.

As outlined above, site‐selective removal of covalently bound functionalities would represent a fundamental leap forward in the functional group structuring and structural control of grafted chemical information. Inspired by the laser‐triggered reduction of GO,^[^ ^20^, ^21^, ^22^ ^]^ we were able to develop a first approach toward this ultimate goal: a laser path‐selective functional group removal and regeneration of pristine graphene with high spatial control (**Scheme** **2**). Similar to the work of Sokolov et al.,^[^ ^22^ ^]^ a laser in the visible spectral range (*λ_exc._* = 532 nm) with high laser powers (*P_L_* ≈ 20 mW) and long irradiation times (*t*) has been chosen. In order to study the effect of high laser power irradiation on functionalized graphene, a sample was prepared in a similar manner to that for the thermally induced defunctionalization.

**Figure 4:**
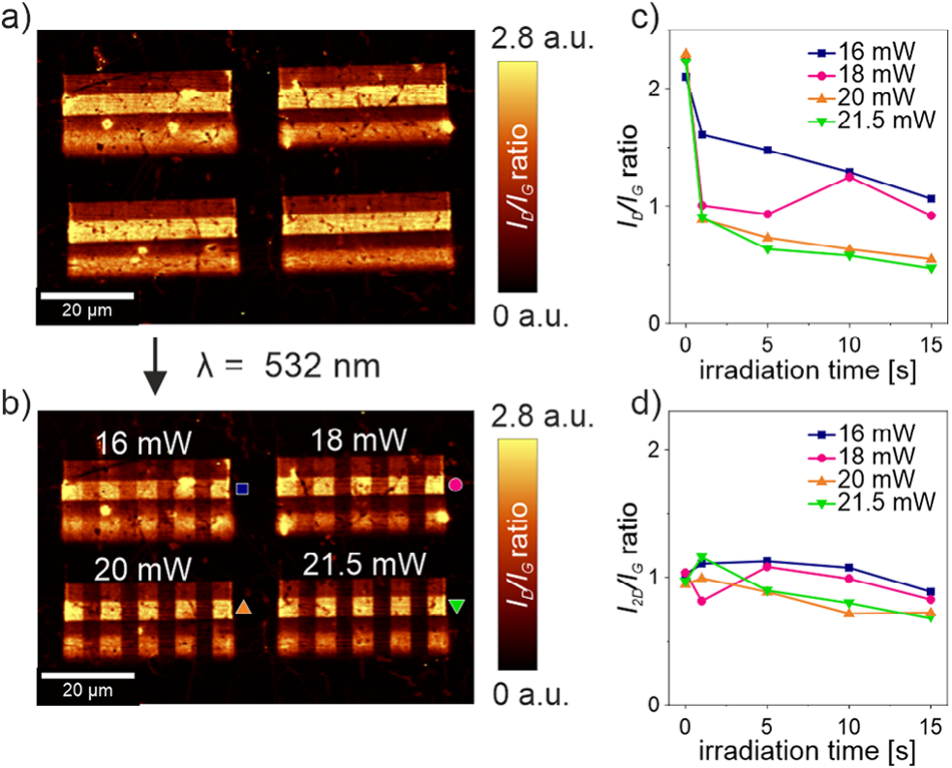
Raman investigation of the laser‐triggered defunctionalization of DBPO functionalized graphene areas with varying degrees of functionalization indicated by the different color brightness of the patterned lines in the *I*_D_ */I*_G_ “readout” mappings. a) Shows the Raman map after the laser‐induced functionalization and b) the Raman map after the laser‐triggered defunctionalization performed perpendicular to the original patterned lines. The laser powers applied for each block are indicated above the patterns, each perpendicular stripe corresponds to a specific irradiation time (see Figures S7 and S8 for further details, Supporting Information). *I*_D_ */I*_G_ ratios c) and *I*_2D_ */I*_G_ ratios d) plotted as a function of applied exposure time and laser power for an exemplary degree of functionalization marked next to the corresponding stripe in b) (see Figures S8–S10 for full Raman investigation, Supporting Information).

For this purpose, a functionalized monolayer graphene with four different degrees of functionalization generated by applying different laser “writing” parameters, arranged as four lines, has been prepared (**Figure** **4**, left). Since we decided to vary the “erasing” parameters for a better understanding of the process, four equal blocks have been prepared, each being laser “erased” with one fixed *P_L_* and four different irradiation times (*t* = 1, 5, 10, 15 s) (see Figure S7 for details, Supporting Information). Figure 4 shows the *I*_D_ */I*_G_ ratio Raman “readout” maps before and after the laser “erasing.” Prior to the high laser power irradiation, the different functionalized areas can be clearly observed, presenting a homogeneous appearance for each area. The selected regions were irradiated with the indicated parameters in a line‐based spectral scanning mode, which allows an even energy distribution over the selected area, representing the laser “erasing” step. SRM mapping was then performed again to “read out” the modified chemical information. In this “readout” Raman mapping, clear differences between the high laser power irradiated areas and the non‐irradiated functionalized areas can be observed (for detailed spectral information, see Figures S8–S10, Supporting Information). Depending on the applied laser parameters, the *I*_D_/*I*_G_ ratio decreased to different extents: a slight *I*_D_/*I*_G_ decrease in the case of *P_L_* = 16 mW and an *I*_D_/*I*_G_ decrease close to the unmodified background level in the case of the highest laser power (*P_L_* = 21.5 mW).

It should be noted that according to the respective *I*_2D_/*I*_G_ Raman maps (Figure S8, Supporting Information), a slight decrease in the *2D* band intensity is observed in the irradiated areas. The evolution of the *I*_D_ */I*_G_ ratio as well as the *I*_2D_/*I*_G_ ratio as a function of the applied laser power (*P*_L_) and irradiation time is summarized in Figure 4c,d (the corresponding average Raman spectra are shown in Figure S9, Supporting Information). The data clearly show that a successful laser path‐selective defunctionalization of graphene can be achieved at high laser powers.

Kelvin Probe Force Microscopy (KPFM) measurements were performed to gain further insight into the high laser power irradiated areas (see Figure S11, Supporting Information). The KPFM images show a significant drop in surface potential for these, while the functionalized area is indistinguishable from the unmodified background due to the electronically neutral behavior of the attached phenyl rings with respect to the surrounding graphene. Note that these changes in 2D band intensity and surface potential occur not only when covalently functionalized areas are irradiated, but can also be observed when the same conditions are applied to unmodified pristine graphene (see reference experiment in Figures S12–S15, Supporting Information). This leads to the assumption that these changes are not directly correlated with the removal of functional groups and are most likely due to side reactions that graphene can undergo under ambient conditions, triggered by high laser power irradiation. A possible explanation for the observed decrease in surface potential could be the attachment of oxo species from the environment; a detailed investigation of this phenomenon is currently underway in our laboratory.

We suggest that the laser‐induced detachment of covalently bound entities is due to a photothermal process. This is in line with the laser‐triggered reduction of GO,^[^ ^20^ ^]^ where a thermal effect,^[^ ^23^ ^]^ a photochemical effect,^[^ ^24^ ^]^ or a combination of both can be discussed.^[^ ^25^, ^26^ ^]^ According to the previous studies on “laser reduction of GO,” a photo‐reduction is only possible for a wavelength shorter than 390 nm,^[^ ^27^ ^]^ which leads to the conclusion that photothermal effects are responsible for the observed laser‐triggered reduction above 390 nm.^[^ ^20^ ^]^ Thermal reduction of GO can occur between and 230 °C,^[^ ^28^, ^29^ ^]^ which is in a similar range as we presented earlier for our functionalized graphene.

**Figure 5:**
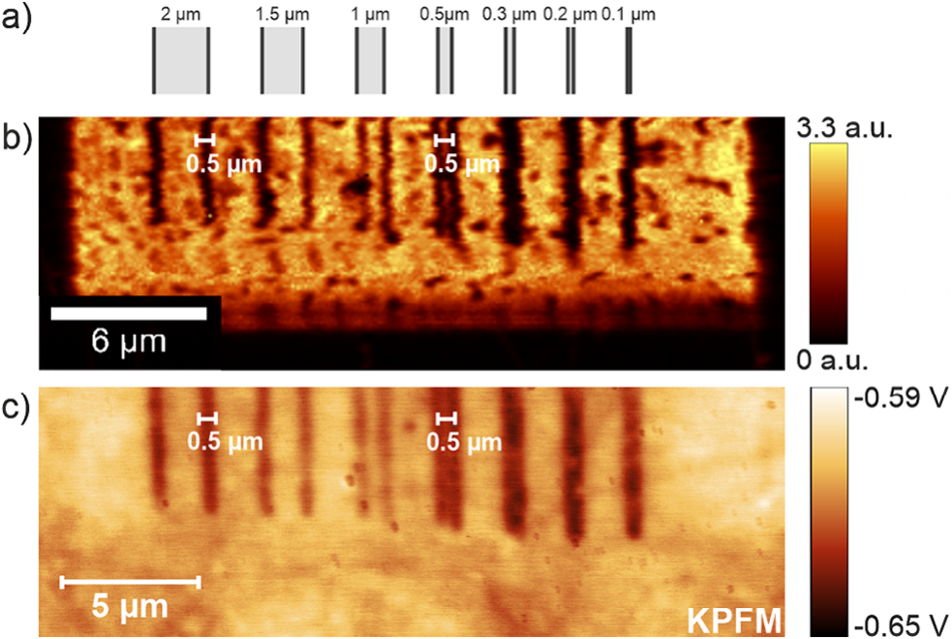
a) Schematic representation of the line scan pattern used to investigate the smallest possible pattern size achievable with the laser “erasing” approach. b) “Readout” Raman *I*_D_ */I*_G_ mapping for the line scan pattern generated with 20 mW. c) KPFM image of the same area (see Figures S16–S18 for further details, Supporting Information).

We have recently shown that the laser‐based covalent functionalization of graphene using DBPO as a photoactive precursor can be performed with a high degree of lateral precision,^[^ ^10^ ^]^ and this leads directly to the question of whether laser‐triggered functional group “erasure” can achieve a similar lateral resolution. To this end, a laser‐path‐guided “erasing” pattern of line pairs with decreasing spacing was applied to phenyl‐functionalized monolayer graphene (**Figure** **5**). The distance between the lines generated in a line scan mode, which allows multiple single spectra along a line with predefined irradiation parameters, was set to distances starting from 2 µm, going down to 0.1 µm.

Figure 5b and c show the *I*_D_ */I*_G_ Raman mapping and the KPFM image obtained after the applied laser “erasing” sequence. Both show distinguishable line pairs down to a line pair separation of 0.5 µm, resulting in a line width of 0.5 µm, which can be considered as the smallest possible pattern size for the laser‐triggered defunctionalization. This agrees well with the reported smallest pattern size of 0.55 µm for laser‐reduced GO.^[^ ^24^ ^]^

By combining the functional group laser “writing” with the site‐selective laser‐based defunctionalization presented here, it becomes possible to not only introduce new information into the graphene lattice in a highly patterned manner, but also to increase the functional group structuring on the graphene surface by regio‐selectively “erasing” specific parts of the covalently attached moieties. Again, a fundamental requirement is the ability to successfully “rewrite” laser “erased” regions in the monolayer graphene lattice, providing full “write”/“read”/“erase” cycles for the covalent 2D storage of chemical information.

**Scheme 3:**
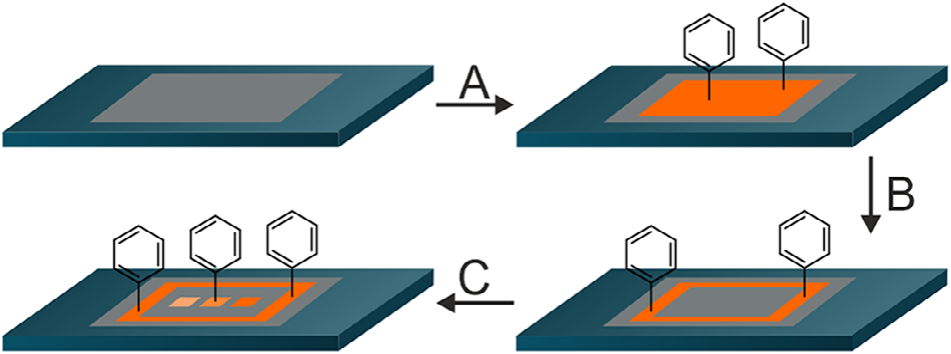
Schematic representation of A) the first laser “writing” applied, B) the performed laser “erasing” and C) the laser‐triggered refunctionalization of the beforehand laser defunctionalized area.

To prove the viability of our concept, a large area of graphene was functionalized with DBPO using the laser “writing” process (**Scheme** **3**), followed by a laser‐path‐guided defunctionalization of extended areas, leaving only a frame‐like structure of functionalized graphene around. The sample was then recoated with DBPO, and a new laser “writing” sequence was applied to the previously erased area (see Figure S19 for details, Supporting Information).

In the second “writing”, different laser parameters were applied in order to obtain fundamental insights into the refunctionalization ability of laser “erased” graphene. For a better comparison of the refunctionalization in the “erased” region with the regular laser‐triggered functionalization of pristine graphene, a reference “writing” pattern with the same laser “writing” parameters was generated next to the initial region.

**Figure 6:**
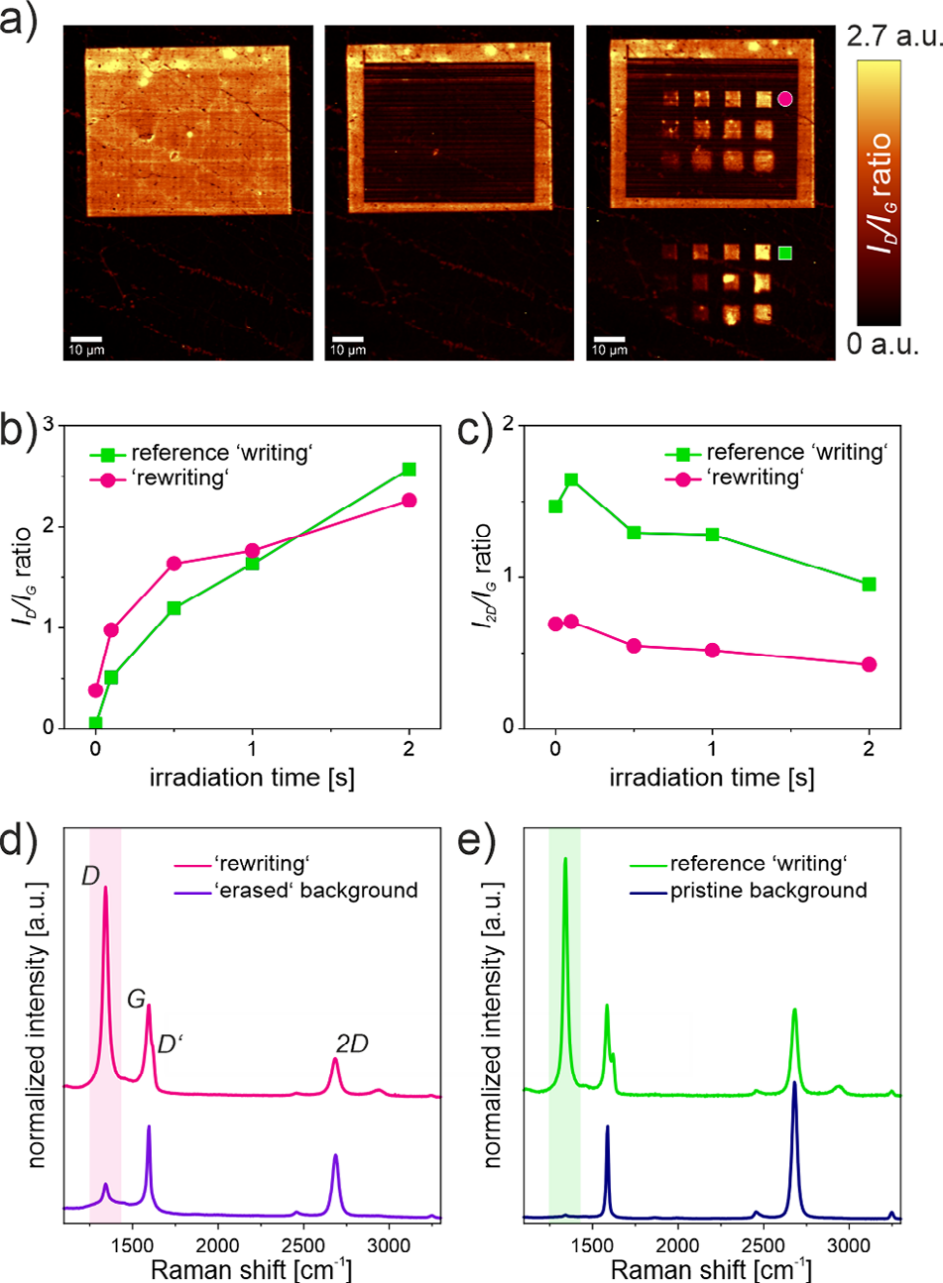
a) *I*_D_ */I*_G_ “readout” Raman mappings after the stepwise functionalization of graphene with DBPO (left), local “erasing” (center), and “rewriting” (right) ‐ see Supporting Information for applied parameters. Corresponding b) *I*_D_/*I*_G_ and c) *I*_2D_/*I*_G_ plot. d) Comparison of the Raman spectra in the “erased” and “rewritten” area (*P_L_ *= 0.5 mW). e) Comparison of the Raman spectra of the unfunctionalized pristine graphene region and the covalently functionalized reference region (*P_L_ *= 0.5 mW) (see Figures S19–S22 for further details, Supporting Information).

**Figure** **6** shows the *I*_D_ */I*_G_ Raman mapping after each step, illustrating the precise functionalization by laser “writing” and the well‐controlled laser‐triggered defunctionalization in the first two steps.

After the last step, new distinct domains with increased *I*_D_ */I*_G_ ratios emerge in the laser “erased” area. According to the color scale, the degrees of functionalization of the refunctionalized areas are highly comparable to those of the reference “writing” (further information in Figures S20–S22, Supporting Information). These findings are further supported by the evolution of the respective *I*_D_ */I*_G_ ratios for the different laser “writing” parameters (Figure 6b), which show a correlated increase in *D* band intensity with increasing irradiation time for a fixed *P_L_*, the corresponding information on the development of the *2D* band is given in Figure 6c. The differences in the measured *2D* intensity values (reference “writing” vs “rewriting”) may be attributed to an absolute decrease in *2D* band intensity found after the laser “erasing” procedure, due to the observed changes in the laser “erased” regions (see KPFM discussion above).

**Figure 7:**
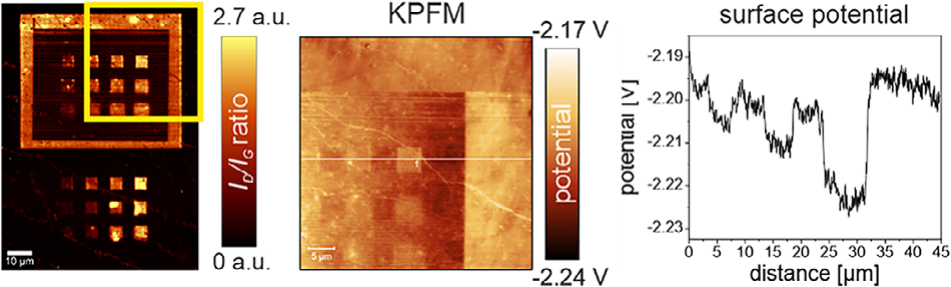
KPFM analysis of stepwise functionalized graphene: *I*_D_ */I*_G_ Raman map indicating the exact location of the KPFM image of the refunctionalized area (left), the corresponding KPFM image (center), and potential profile (right) (see Figures S24–S25, Supporting Information for further details and KPFM image of reference area).

Nevertheless, the normalized mean Raman spectra presented in Figure 6d clearly prove the successful second laser‐triggered functionalization step, carried out on a laser “erased” monolayer graphene region. The generated pattern shows long‐term stability of at least 13 months, supporting the high versatility (Figure S23, Supporting Information). In addition, KPFM measurements (**Figure** **7**) were performed, which further substantiated our results and provided additional interesting findings. As shown before, the laser “erased” region differs from the “non‐erased” area and the corresponding unmodified pristine background by exhibiting a lower surface potential.

Surprisingly, the refunctionalized areas can be clearly distinguished from the surrounding laser “erased” background, due to their higher surface potential. This was unexpected, as the electronic nature of the attached phenyl groups usually does not provide a local difference in surface potential with respect to the graphene background (Figure 7). A possible explanation for this behavior could be that although attached phenyl rings do not influence the surface potential of unmodified graphene, these phenyl moieties could show an electron‐donating effect on partially oxidized graphene regions, as probably generated during the laser‐based “erasing” process. This suggests that the electron‐withdrawing nature of the attached oxo‐moieties is effectively balanced by the introduction of phenyl groups, which exhibit a mildly electron‐donating character relative to the partially oxidized graphene, during refunctionalization. This would explain the dependency of the surface potential reached on the degree of functionalization and why even the surface potential of highly refunctionalized graphene is still lower compared to non‐modified graphene.

**Figure 8:**
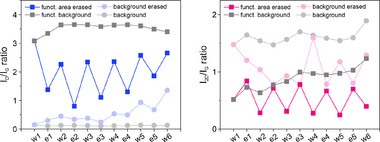
Evolution of the *I*_D_/*I*_G_ and *I*_2D_/*I*_G_ ratio in a multi‐step procedure of laser “erasing” and laser‐induced “rewriting” with DBPO (see Figures S26–S28 for further details, Supporting Information).

After demonstrating the efficient “rewriting” on laser‐“erased” graphene, we again conducted a multi‐step “erasing” and “rewriting” process. This allowed us to confirm the high durability of the material, performing at least five cycles (**Figure** **8**).

**Figure 9:**
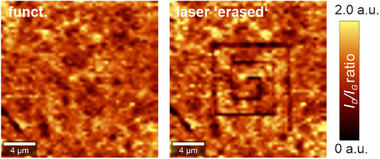
*I*_D_ */I*_G_ Raman mappings of reductively functionalized graphene before (left) and after (right) local defunctionalization generated by the presented laser “erasing” procedure (see Figure S29 for experimental details, Supporting Information).

To illustrate the broad applicability of our presented laser “erasing” concept, we have extended our investigation to include a covalently functionalized graphene sample obtained via a classical, reductive functionalization approach. These results, summarized in **Figure** **9**, prove the high versatility of our procedure and pave the way for structuring covalent functionalization after a wafer scale functionalization reaction.

## 3 Conclusion

Our present study demonstrates that thermally defunctionalized graphene can be reused for subsequent laser‐triggered functionalization, allowing for “erasing” and reintroducing of chemical information within the graphene lattice in multiple cycles. Furthermore, we have pioneered an innovative laser‐triggered defunctionalization sequence that enables the site‐selective removal of covalently bound entities in individually targeted areas with high precision and in 2D patterns down to 0.5 µm. This represents a complementary, reverse 2D patterning approach. Beyond laser‐guided functionalization, our method now enables highly precise and site‐selective removal of covalent groups. This unlocks unprecedented flexibility in controlling the shape, area, and lateral dimensions of the final 2D pattern. In addition, the tunable, laser‐path “erased” areas can be subsequently functionalized, providing a complete cycle of generation, storage, and selective removal of chemical information localized in graphene that can be applied multiple times, representing a major step toward application in organic information storage.

## Conflict of Interest

The authors declare no conflict of interest.

## Supporting information



Supporting Information

## Data Availability

The data that support the findings of this study are available at Zenodo at http://doi.org/10.5281/zenodo.16947058.
