# Supplementary material for: Laser‐Induced Covalent Defunctionalization of Graphene — Precise Patterning and Site‐Selective Removal of Functional Groups
Source: Adv Sci (Weinh). 2025 Aug 30;12(43):e11481. doi: 10.1002/advs.202511481 (PMC12631891; doi:10.1002/advs.202511481)
Supplement: Supplementary file 1 — Supporting Information [file ADVS-12-e11481-s001.pdf]

SUPPORTING INFORMATION

Laser-Initiated Covalent Defunctionalization of Graphene – Precise Patterning and Site-Selective Removal of Functional Groups

Tamara Nagel, Kevin Gerein, Frank Hauke and Andreas Hirsch*

Department of Chemistry and Pharmacy & Center of Advanced Materials and Processes (ZMP), Friedrich-Alexander-Universität Erlangen-Nürnberg, Nikolaus-Fiebiger-Str. 10, 91058 Erlangen, Germany

Table of Contents

1. Instrumental Section 2

1.1. Raman Spectroscopy 2

1.2. Scanning Probe Microscopy 3

2. Experimental Section 3

2.1. Materials and Chemicals 3

2.2. Sample Preparation 3

3. Data Section 6

3.1. Temperature Induced Defunctionalization 6

3.2. Local Refunctionalization on Thermally Defunctionalized Graphene 8

3.3. Multi-Step Local Refunctionalization on Thermally Defunctionalized Graphene 9

3.4. Laser-Initiated Defunctionalization 12

3.5. Reference Experiment: High Laser Power Irradiation on Covalently Functionalized Graphene and the Non-Modified Background 15

3.6. Laser-Induced Defunctionalization – Lateral Dimensions 19

3.7. Rewriting after 'Erasing' 20

3.8. Long-Term Stability 25

3.9. Multiple Cycles 'Rewriting' after 'Erasing' 26

3.10. Laser 'Erasing' on Wafer Scale Functionalized Graphene 27

1. Instrumental Section

1.1. Raman Spectroscopy

The laser 'writing' and 'erasing' procedures as well as Raman spectroscopy for characterization were performed on a WITec alpha300R confocal microscope equipped with a motorized xyz table (Δxy_{min} = 10 nm, Δz_{min} = 30 nm) and a high-sensitivity CCD detector (1,024 x 127, 80 spectra per second). The Raman system was coupled to an optical microscope operating both in bright and dark field, as well as an internal calibration source. The setup was controlled by the WITec Control software, equipped with EasyLink, facilitating control over spatial coordinates, and TruePower function, enabling a distinct adjustment of the laser power (P_L) on the sample surface with an accuracy of <0.1 mW, ensured by laser power measurements in the optical fiber. This has also been confirmed by an external P_L calibration. Measurements were conducted with a 100x Zeiss EC "Epiplan-Neofluar" DIC (NA = 0.90) and for temperature dependent measurements with a LD 50x Zeiss EC "Epiplan-Neofluar" DIC (NA = 0.5) objective and grating of 600 grooves/mm. All acquired data were evaluated with the WITec Project software. All experiments without further notice were carried out with a laser excitation wavelength of λ = 532 nm (DPSS laser with a maximum P_L of 30 mW). The laser was coupled to a custom long-wavelength filter prior to coupling to the Raman optics. For temperature dependent Raman measurements the Raman setup was coupled with a THMS600 Linkam Heating and Freezing stage

(Temperature range -196 °C – 600 °C) controlled by the Link software, allowing heating rates between 0.01 °C/min and 150 °C/min.

1.2. Scanning Probe Microscopy

Scanning probe microscopy was carried out using a Bruker Dimension Icon 3 microscope in AM-KPFM mode (amplitude modulated kelvin probe force microscopy). Bruker SCM-PIT-V2 probes (platinum-iridium coating) on antimony doped Si cantilevers were used to obtain the KPFM images with 512 x 512 pixels or 1024 x 1024 pixels resolution (scan rate: 0.2 Hz). The operative software was NanoScope 9.4 and the received data were evaluated with Gwyddion.

2. Experimental Section

2.1. Materials and Chemicals

CVD graphene was purchased from ACS Materials Inc. as trivial transfer graphene with PMMA coating. Si/SiO_2 wafers (300 nm thick silicon oxide layer) were obtained from Fraunhofer e.V. (Germany) and cut to 0.5 x 0.5 mm substrates. DBPO was bought from Merck Sigma-Aldrich Chemie GmbH. All other chemicals and solvents were purchased from Merck Sigma-Aldrich Chemie GmbH, Carl Roth GmbH, and VWR International GmbH and, if not stated otherwise, used with no further purification.

2.2. Sample Preparation

Transfer of CVD Graphene on Si/SiO_2 Substrate:[1] The target substrate was cleaned beforehand by iterative spin coating with acetone (99 %, HPLC grade) and isopropanol (99 %, HPLC grade). The CVD graphene/PMMA film located on the carrier material was moistened carefully to loosen the graphene-carrier bond. Thereafter, it was released into double distilled water, letting the graphene float on the surface. To divide the graphene into smaller pieces the floating sheet was picked up using a filter paper, cut with sharp scissors, and immediately re-floated on the double distilled water surface. Afterwards, the pre-cleaned substrate (Si/SiO_2 wafer) was placed in the water underneath the cut floating film and cautiously pulled out of the water to position the film on the surface. Then, the sample was dried and heated to 130 °C for one hour to remove any residual water trapped under the graphene lattice. The protective PMMA layer was then gently removed using acetone vapor at 60 °C for 2 hours.

General 'Write'/'Read' Functionalization Procedure:[1] In a first step, a suitable area of pristine graphene on a Si/SiO_2 substrate was selected on the sample by optical microscopy and pre-characterized by Raman spectroscopy to ensure the appropriate quality of the graphene. The selection criteria were mainly based on an optically mostly defect free surface, combined with a regular

graphene Raman spectrum as well as some specific sample features such as holes, wrinkles, multilayer islands or selective surface contamination as natural markers. To ensure that the functionalized areas can be precisely localized again in the further course of the investigations, the sample was mounted on the automated xy table with the edges aligned as parallel as possible to the axes. Coordinates were set and optical images and stitched optical images of the selected area and the entire sample were taken. This is a very important step, as the covalent functionalization of graphene introduced later does not change the optical appearance of graphene, making it impossible to find the laser functionalized area without detailed process planning and recording.

Afterwards, the functionalization reagent was applied on the graphene surface using a spin coating procedure. For this purpose, a solution of 10^{-3} M DBPO in diethyl ether was prepared and used at the same day to ensure the stability. Subsequently, the clean substrate bound graphene was spin coated with 1 - 3 drops of the DBPO solution with 4,000 rpm for 30 s.

After coating, the prepared specimen was remounted on the automated xy stage with the same orientation as before. Using the previously set coordinates and the optical images of the original measurement, the selected area can be found again. To ensure a sufficient reactivity of the coating, several Raman single point spectra (*e.g.* λ = 532 nm, P_L = 0.5 mW, t = 0.5 s, 10 acquisitions) were performed in the surrounding, which already need to show functionalization of graphene due to an increasing *D* band. For the laser 'writing' functionalization process, a specific pattern was designed in advance, including the laser 'writing' parameters and the dimensions and positions of the areas. These were specified in the WITec Control software. The coordinate system allows the precise positioning of the areas. The laser-triggered covalent graphene functionalization was performed as large area scans in area mapping mode, *i.e.* a line-based spectral scan of the sample is performed, facilitating the local functionalization process while roughly monitoring the reaction with *in situ* Raman information – standard 'writing' parameters: P_L = 0.5 mW, t = 2 s, 0.5 µm step size). In general, the following parameters can be adapted: laser excitation wavelength (λ), laser power (P_L), irradiation time of each point (t), the dimensions in x and y, the distance between points as well as the position according to the coordinate system. Using the area-mapping mode has a great advantage over regular rasterizing of individual spectra. In this mode, the excitation laser is moved continuously over the sample without stopping the laser path at each point, resulting in a more uniform irradiation of the surface and a significantly more homogeneous laser-induced functionalization of the graphene. Prior to each measurement, the laser beam was focused on the sample using the system's oscilloscope mode in an area adjacent to the planned pattern to avoid unintentional side functionalization. After the actual laser patterning procedure, the sample was removed from the Raman stage and the residual coating was washed off by immersing the sample in diethyl ether for 10 minutes, rinsing it with isopropanol,

and drying under pressurized air to prevent any unwanted modification of the graphene lattice during the ‘readout’ process. For the Raman ‘readout’ characterization, the sample was again placed on the automated stage of the Raman setup and the patterned area was identified using the previously set coordinates and the obtained optical images. To confirm the completeness of the washing procedure, several single point Raman measurements were performed using similar parameters as for the reactivity study of the coatings, which must present typical pristine graphene Raman spectra. The Raman ‘readout’ measurement was then performed in an area selected to be several µm larger than the laser functionalized areas. Standard parameters for the Raman ‘readout’ are: λ = 532 nm, P_L = 5 mW, t = 0.5 s or 1 s as acquisition time per point and 0.33 µm as point spacing. This process makes it possible to completely decouple the laser ‘writing’ process from the subsequent Raman ‘readout’ measurement and offers enormous potential for customized multi-functionalization patterns.

General Thermally Induced ‘Erasing’ Procedure: After preparing the sample *via* the described laser ‘writing’ procedure and ensuring the high quality of the covalently functionalized areas by scanning Raman microscopy (SRM) the sample was placed in a Linkam 600 Heating stage under N_2 flow. After relocating the functionalized domains the sample was heated to the first temperature step. When reaching the desired temperature, we waited for 10 minutes before starting the ‘readout’ measurement at this temperature step to ensure thermal equilibrium conditions. Then the ‘readout’ measurement was performed with the 50xLD objective and the indicated ‘readout’ parameters at the chosen temperature. Afterwards, the sample was heated to the next temperature step and the corresponding SRM measurement was performed.

General Laser ‘Erasing’ Procedure: The functionalized samples were prepared using the laser ‘writing’ technique described above with DBPO as the functionalization reagent. After confirmation of the successful covalent functionalization of graphene by Raman spectroscopy, the laser-induced defunctionalization was performed. Therefore, the functionalized areas were located using optical markers as well as the before set coordinates. Afterwards, fast Raman mappings (P_L = 5 mW, t = 0.1 s, 0.5 µm point distance) were used to center the functionalized areas, which allows for a precise positioning of the areas for the subsequent high power laser irradiation. All Raman maps were again performed in area mapping mode and the exact parameters were set as annotated in the corresponding sample description. After high power laser irradiation (λ = 532 nm), the laser-induced defunctionalized sample was analyzed by Raman and KPFM measurements.

General ‘Rewriting’ Procedure: The samples were prepared in a multi-step procedure by laser ‘writing’ and ‘laser erasing’. After ensuring the high quality, the samples were then treated the same way as in

the regular laser 'writing' procedure. This means the samples were coated with DBPO in a spin coating procedure and transferred into the Raman setup. Afterwards, the 'erased' domain was located with the help of prior set coordinates and optical images. The selection of the area in the first laser 'writing' and documentation of the area characteristics is crucial for a good alignment during this multi-step 'writing', 'erasing', and 'rewriting' procedure. Choosing a specific marker in the close surrounding as point zero to allow using relative coordinates in relation to this point is highly recommended. After relocating the area and defining the domains for the 'rewriting', some test single spectra were performed to ensure the quality of the DBPO coating. Then the 'rewriting' was performed applying the given parameters, afterwards the reference 'writing' was performed next to it. After the above-described washing procedure, the sample was then characterized by SRM and KPFM.

3. Data Section

3.1. Temperature Induced Defunctionalization

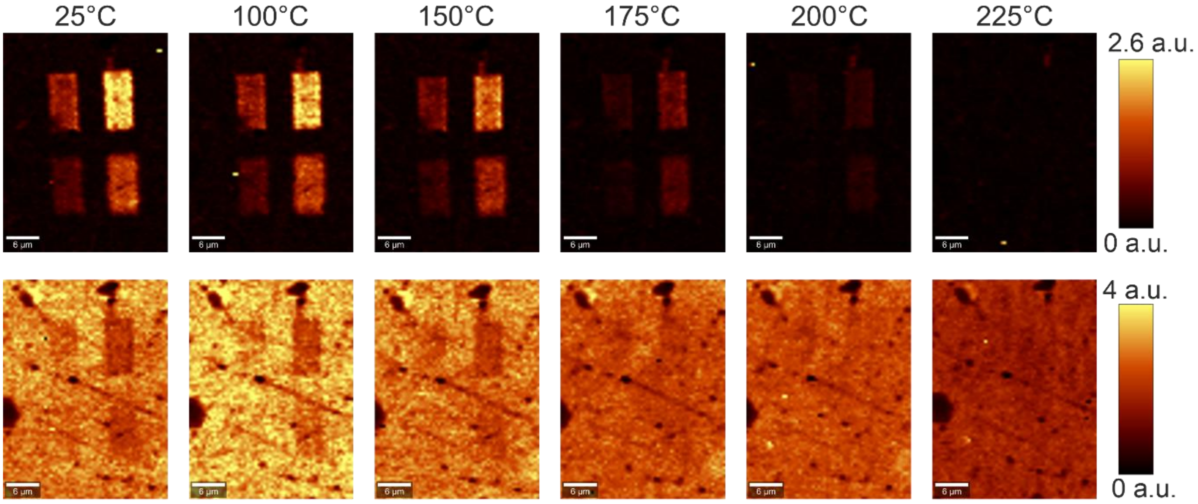

Figure SI 1: I_D/I_G (top) and I_{2D}/I_G (bottom) 'readout' Raman mappings for selective temperatures monitoring the temperature induced defunctionalization by stepwise heating of the sample.
'Writing' parameters: λ = 532 nm, P_L = 0.5 mW (top) and 5 mW (bottom), *t* = 0.5 s (left) and 2 s (right), 5 x 10 µm, 10 x 20 points.
'Reading' parameters: λ = 532 nm, P_L = 10 mW, *t* = 0.5 s to 1 s, 30 x 40 µm, 60 x 80 points; 'readout' Raman measurements at each temperature were started 10 minutes after reaching the desired temperature.

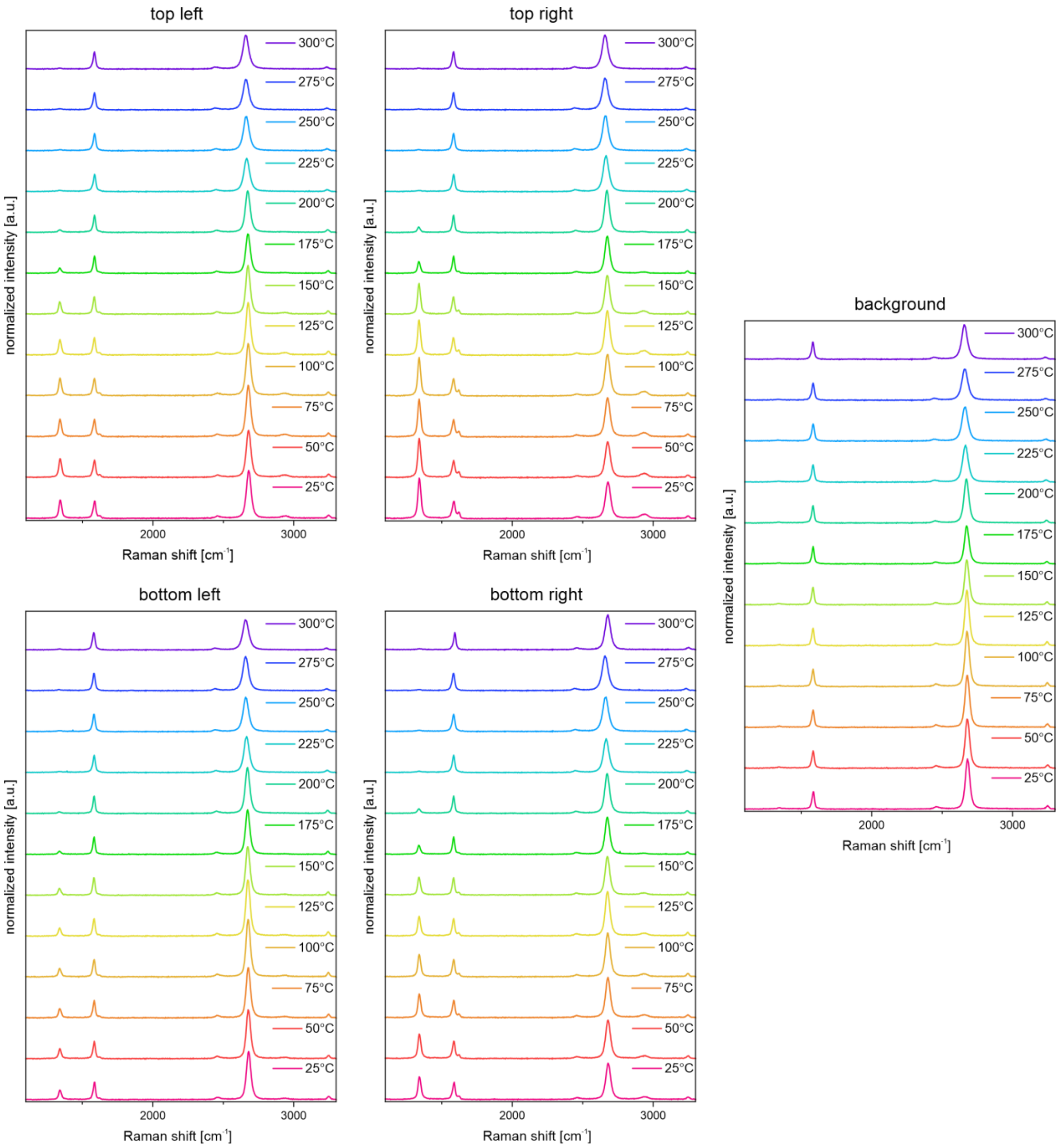

Figure SI 2: Normalized mean Raman spectra monitoring the temperature initiated defunctionalization by stepwise heating.

3.2. Local Refunctionalization on Thermally Defunctionalized Graphene

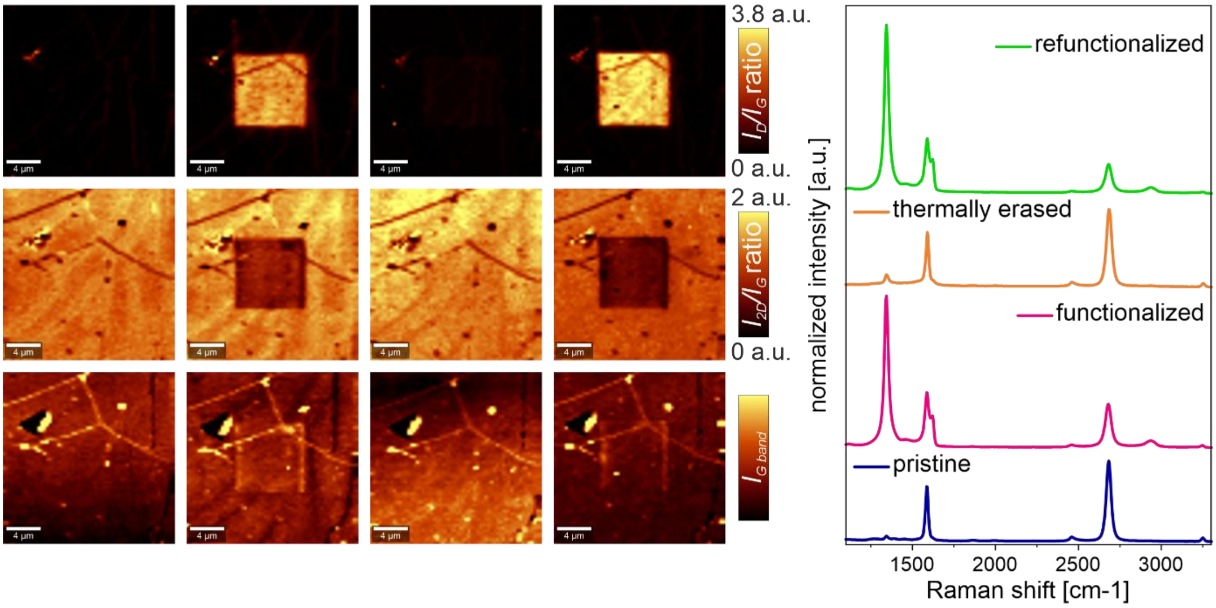

Figure SI 3: I_D/I_G (top), I_{2D}/I_G (middle) and I_G (bottom) 'readout' Raman mappings pristine, after the first laser 'writing', after thermal 'erasing' and after the second laser 'writing' process.
Pristine 'Reading' parameters: λ = 532 nm, P_L = 5 mW, t = 0.5 s, 20 x 20 µm, 60 x 60 points.
'Writing' 1 parameters: λ = 532 nm, P_L = 0.5 mW, t 2 s, 8 x 8 µm, 16 x 16 points.
'Reading' 1 parameters: λ = 532 nm, P_L = 5 mW, t = 0.5 s, 20 x 20 µm, 60 x 60 points.
Thermal heating in Linkam heating stage, 3 h, 225 °C.
'Reading' 2 parameters: λ = 532 nm, P_L = 5 mW, t = 0.5 s, 20 x 20 µm, 60 x 60 points.
'Writing' 2 parameters: λ = 532 nm, P_L = 0.5 mW, t 2 s, 8 x 8 µm, 16 x 16 points.
'Reading' 3 parameters: λ = 532 nm, P_L = 5 mW, t = 0.5 s, 20 x 20 µm, 60 x 60 points.

3.3. Multi-Step Local Refunctionalization on Thermally Defunctionalized Graphene

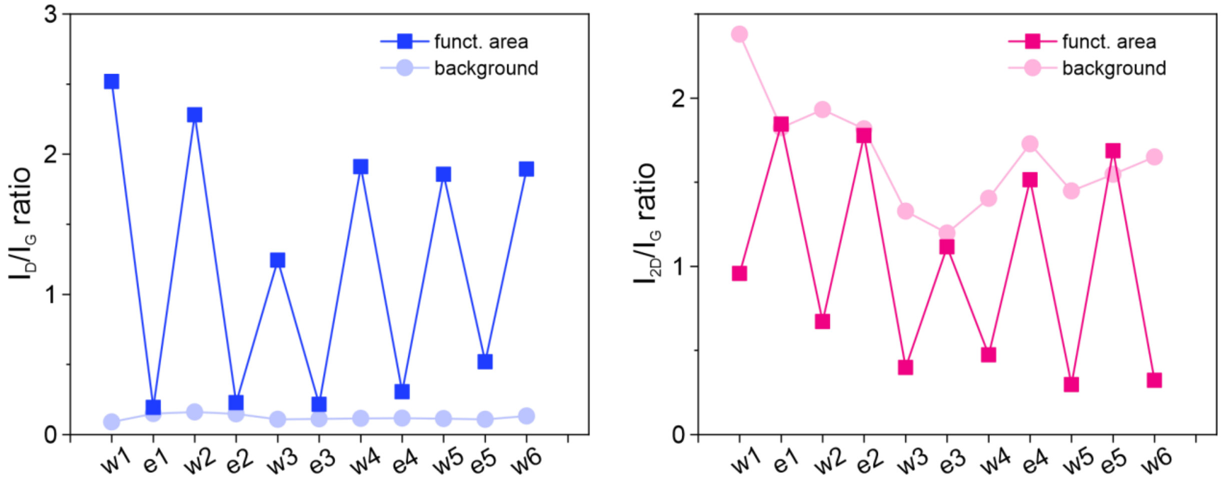

Figure SI 4: I_D/I_G (left) and I_{2D}/I_G (right)ratios for a multi-cycle procedure of 'writing' and 'erasing' covalent functionalization of graphene with DBPO in a laser based 'writing' step (w) and a thermally induced 'erasing' step (e).

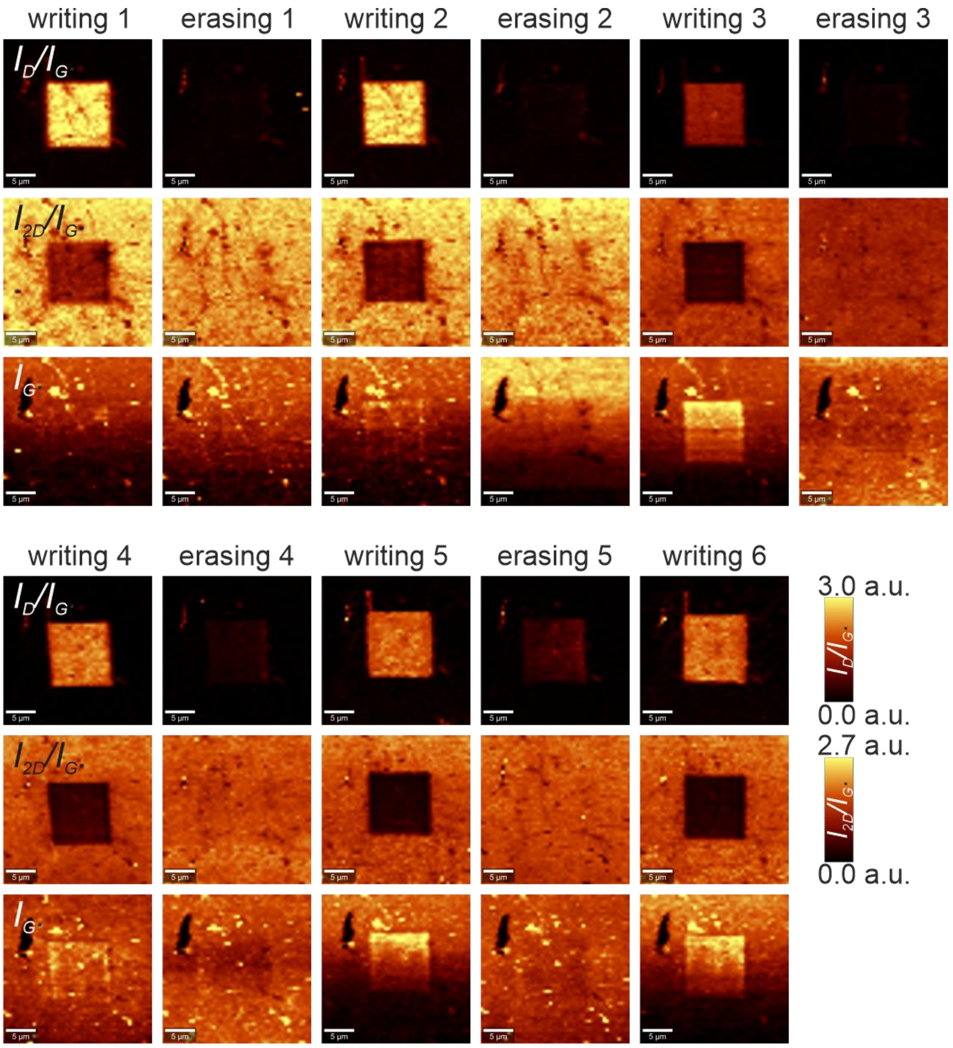

Figure SI 5: ID/IG and I2D/IG ratio as well as g band mapping after each step.

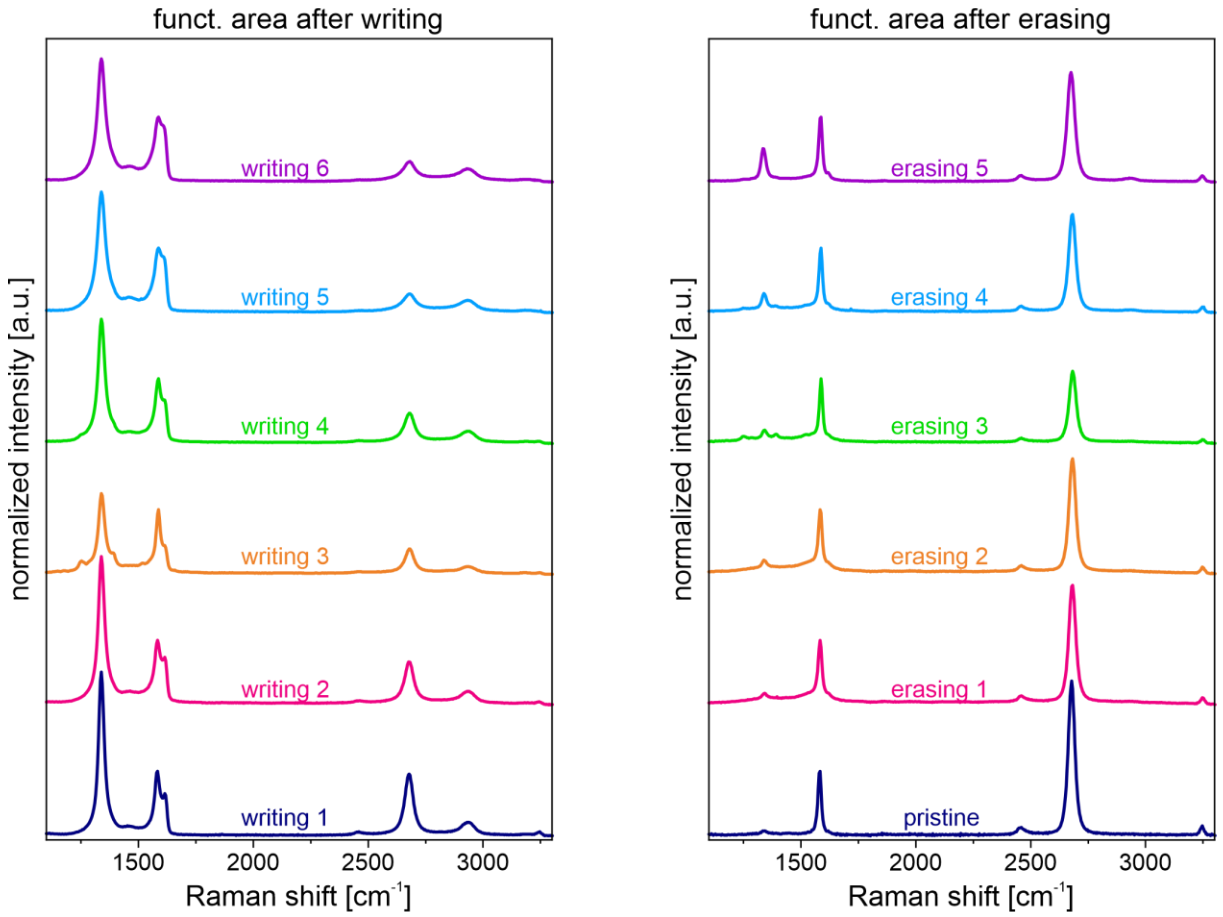

Figure SI 6: Normalized mean Raman spectra of the functionalized area after each step.

3.4. Laser-Initiated Defunctionalization

In the following, the laser-induced defunctionalization of functionalized graphene is investigated in detail.

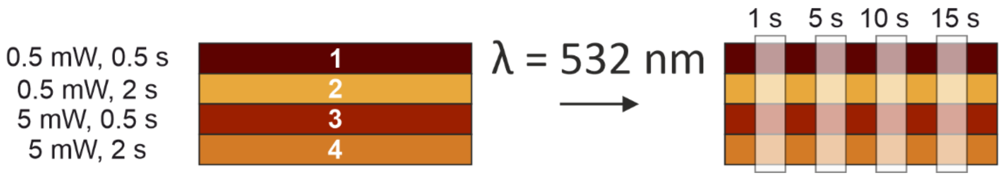

Figure SI 7: Schematic representation of the applied pattern structure to investigate the spatially resolved laser-induced defunctionalization showing the initial writing conditions (left) and the laser-induced defunctionalization irradiation times. This pattern is then applied in four blocks allowing for the examination of four different laser powers.

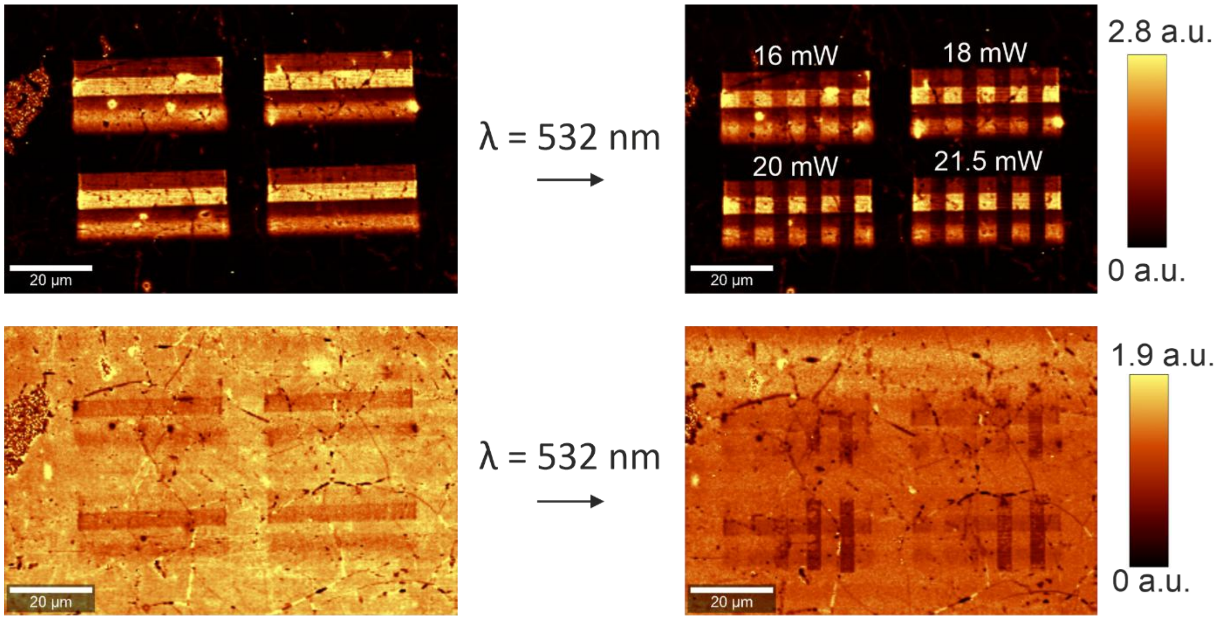

Figure SI 8: I_D/I_G (top) and I_{2D}/I_G (bottom) readout Raman mappings of the functionalized area before (left) and after (right) laser-induced defunctionalization with different parameters.
'Writing' parameters: λ = 532 nm, P_L = 0.5 mW and 5 mW, t = 0.5 s to 2 s, 36 x 4 µm, 72 x 8 points;
First 'reading' parameters: λ = 532 nm, P_L = 5 mW, t = 0.5 s, 100 x 60 µm, 300 x 180 points.
'Erasing' parameters: λ = 532 nm, P_L = 16 mW to 21.5 mW, t = 1 s to 15 s, 4 x 18 µm, 8 x 36 points.
Second 'reading' parameters: λ = 532 nm, P_L = 5 mW, t = 0.5 s, 100 x 60 µm, 300 x 180 points.

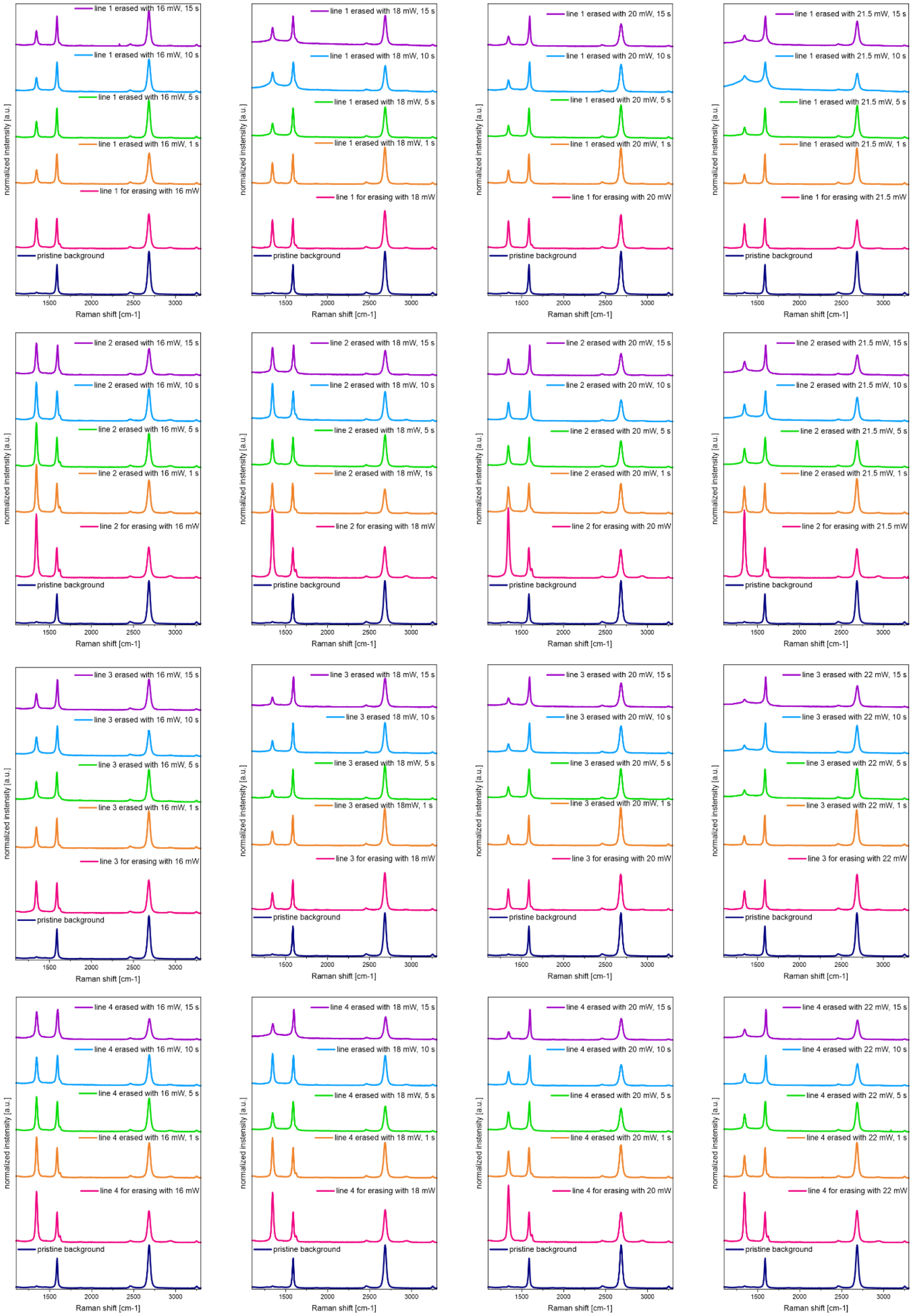

Figure SI 9: Normalized mean Raman spectra of the laser-induced defunctionalized areas.

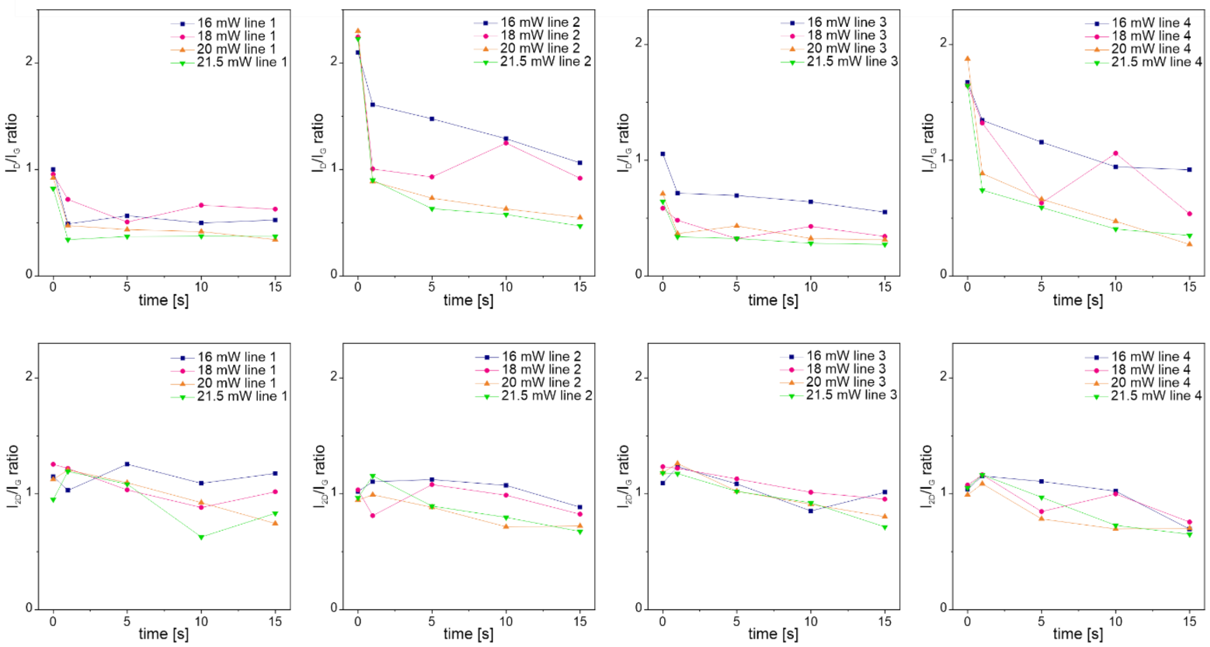

Figure SI 10: Dependency of the I_D/I_G ratio (top) and I_{2D}/I_G ratio (bottom) on the applied irradiation time and laser power for the laser-induced defunctionalization of covalently functionalized graphene.

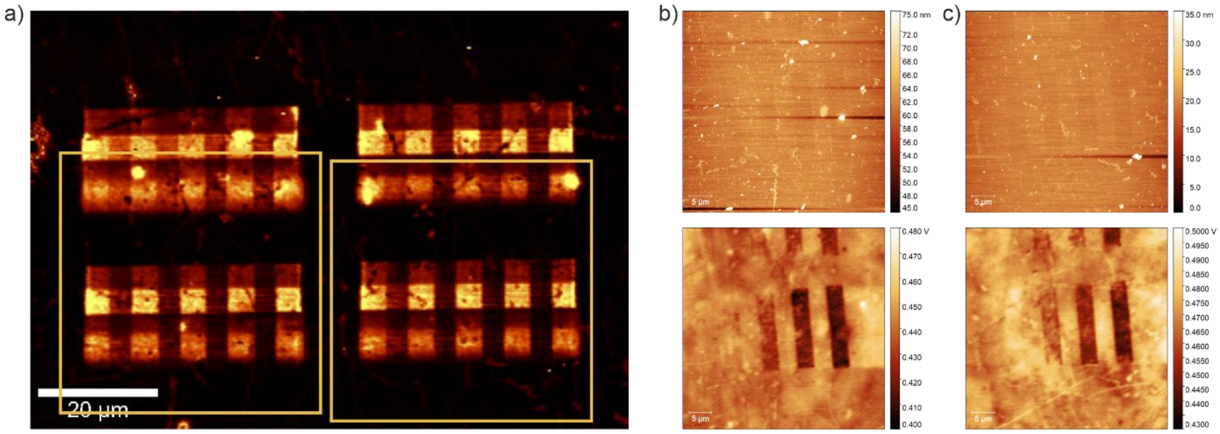

Figure SI 11: (a) Raman map showing the I_D/I_G ratio of the laser-induced defunctionalization of graphene with two squares indicating the exemplary areas of AFM (top) and KPFM (bottom) images presented in (b) (left) and (c) (right).

The high power laser irradiation of covalently functionalized graphene on surface leads to a slight decrease in height of approximately 1 nm compared to the non-irradiated area, while laser reduction of GO tends to show an decrease in height of several tenths of µm.[2] This very different behavior is probably due to the unequal sample structure of a monolayer of graphene on a substrate compared to bulk GO material.

3.5. Reference Experiment: High Laser Power Irradiation on Covalently Functionalized Graphene and the Non-Modified Background

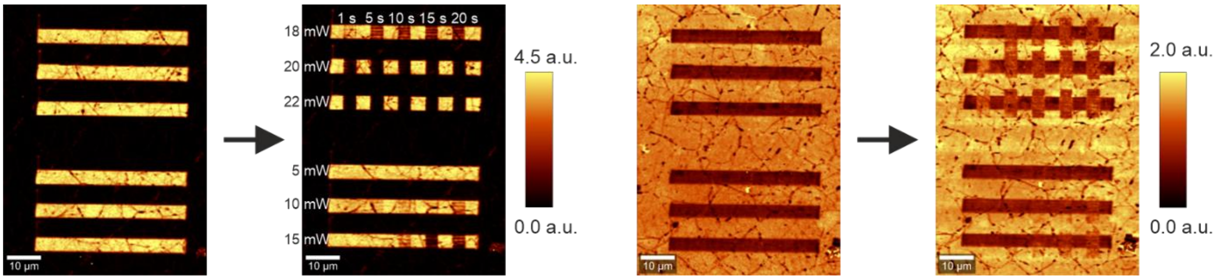

Figure SI 12: I_D/I_G (left) and I_{2D}/I_G (right) readout Raman mappings of the area before and after high laser power irradiation of covalently functionalized graphene as well as non-modified graphene with different parameters.
'Writing' parameters: λ = 532 nm, P_L = 0.5 mW, t = 2 s, 44 x 4 µm, 88 x 8 points.
First 'reading' parameters: λ = 532 nm, P_L = 5 mW, t = 1 s, 60 x 80 µm, 180 x 240 points.
'Erasing' parameters: λ = 532 nm, P_L = 5 mW to 22 mW, t = 1 s to 20 s, 4 x 8 µm, 8 x 16 points.
Second 'reading' parameters: λ = 532 nm, P_L = 5 mW, t = 1 s, 60 x 80 µm, 180 x 240 points.

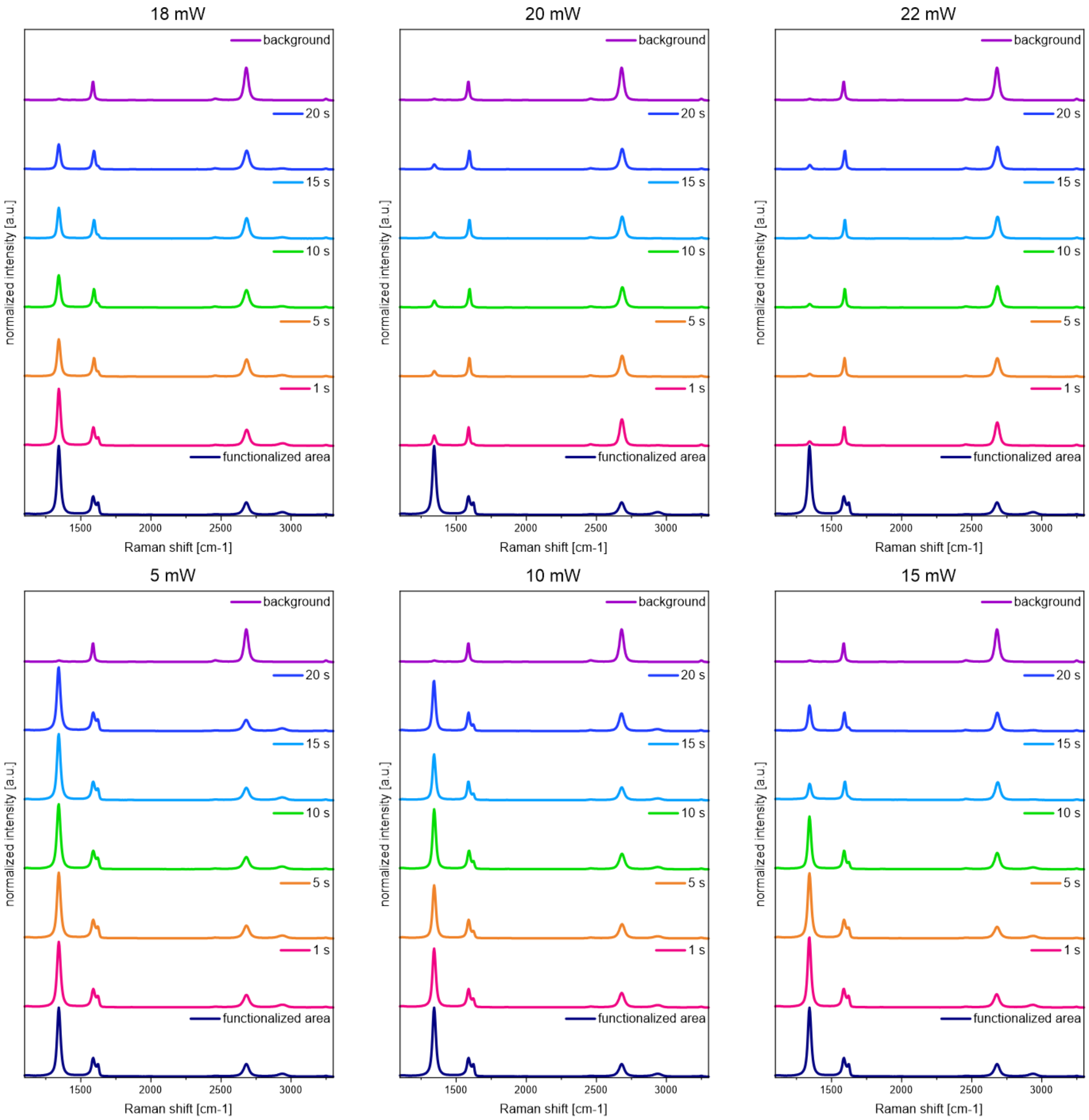

Figure SI 13: Normalized mean Raman spectra of the high laser power irradiated functionalized graphene.

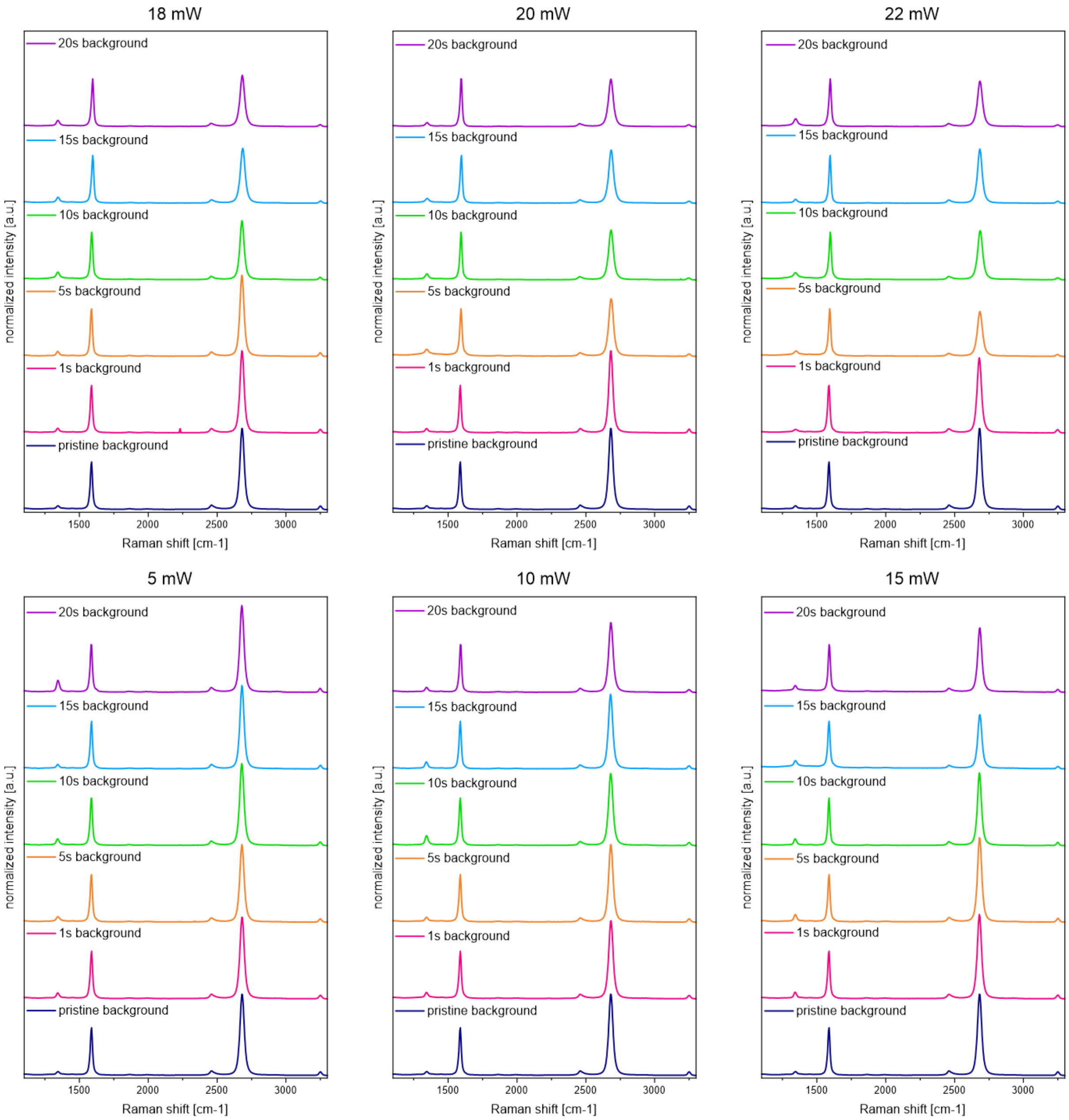

Figure SI 14: Normalized mean Raman spectra of the high laser power irradiated non-modified graphene.

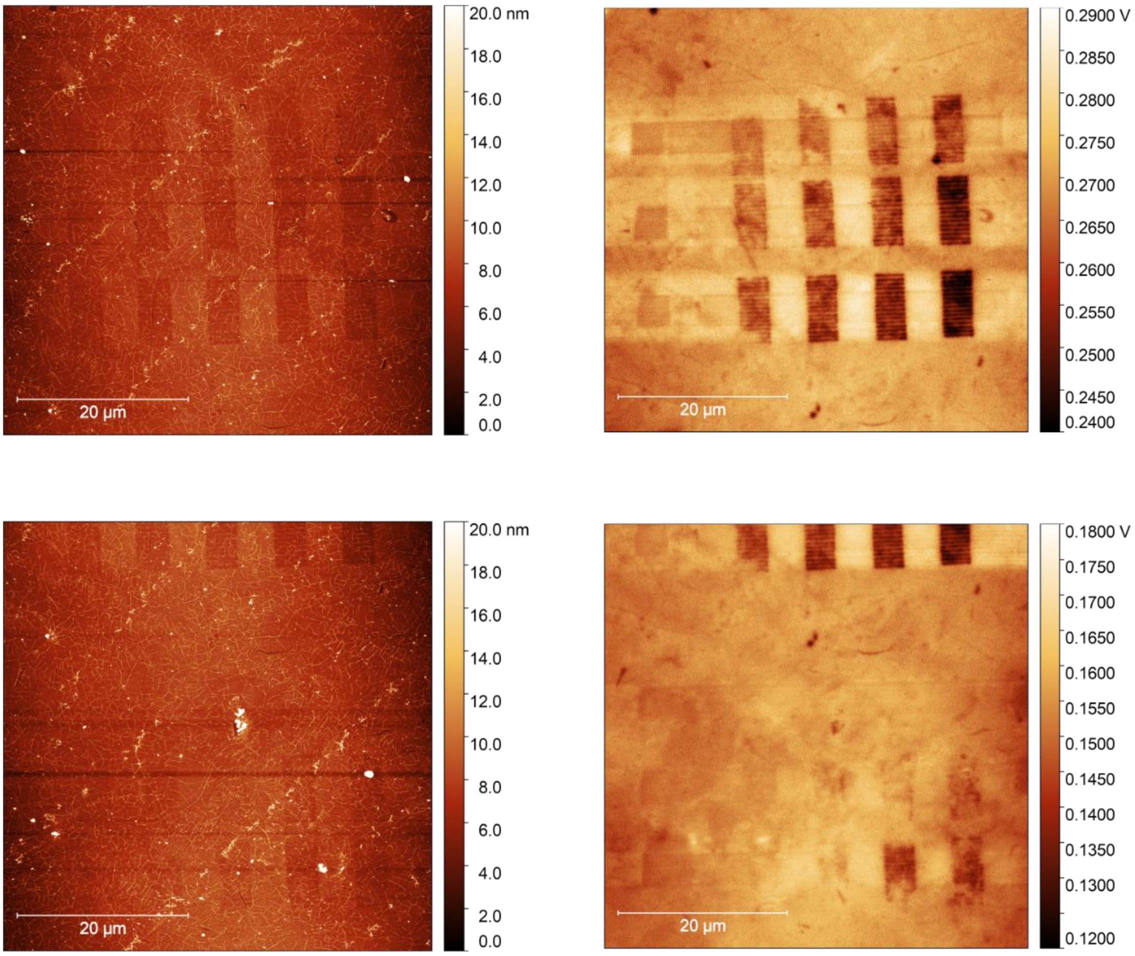

Figure SI 15: AFM and KPFM images of the high laser power irradiated areas of covalently functionalized graphene (top) and non-modified graphene (bottom).

3.6. Laser-Induced Defunctionalization – Lateral Dimensions

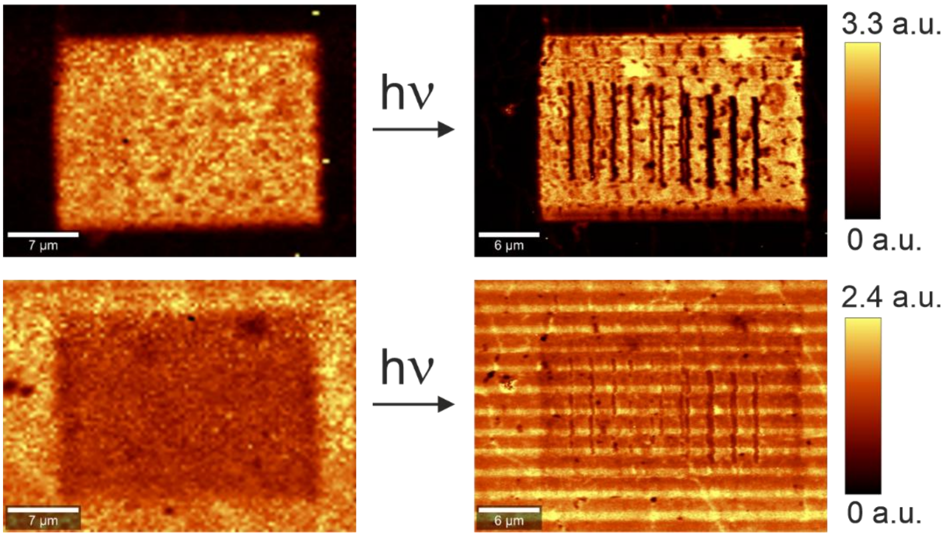

Figure SI 16: I_D/I_G (top) and I_{2D}/I_G (bottom) readout Raman mappings of the functionalized area before (left) and after (right) laser-induced defunctionalization - determination of the minimal lateral dimensions.
'Writing' parameters: λ = 532 nm, P_L = 5 mW, t = 2 s, 25 x 18 µm, 50 x 36 points.
First 'reading' parameters: λ = 532 nm, P_L = 5 mW, t = 1 s, 35 x 25 µm, 105 x 75 points.
'Erasing' parameters: Line scan mode λ = 532 nm, P_L = 20 mW, t = 10 s, 10 µm, 50 points.
Second 'reading' parameters: λ = 532 nm, P_L = 5 mW, t = 0.5 s, 35 x 25 µm, 350 x 250 points.

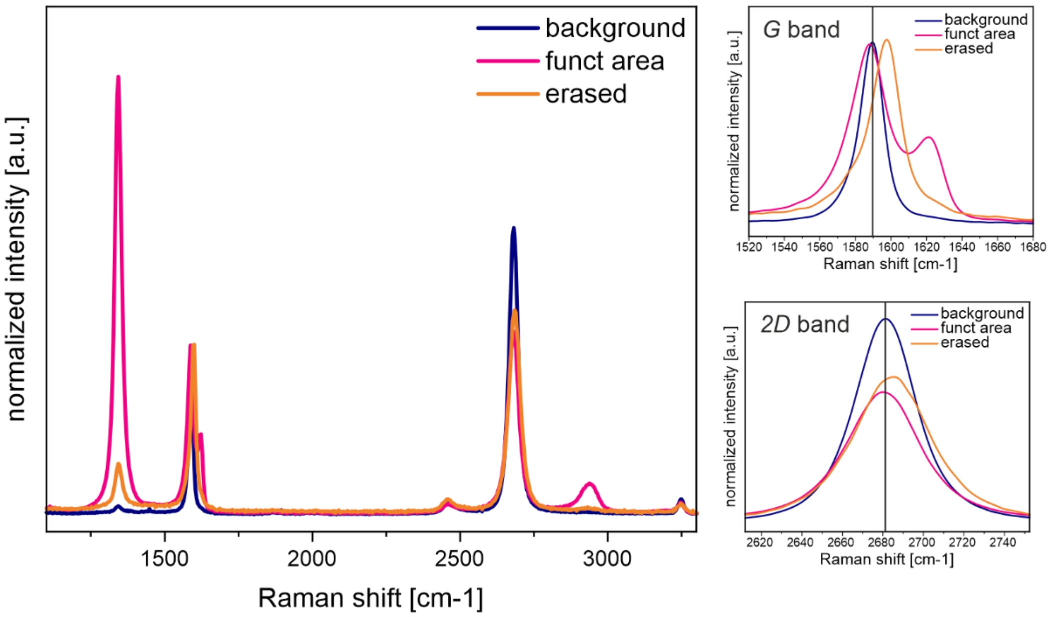

Figure SI 17: Normalized mean Raman spectra of the non-functionalized background (blue), the functionalized area (pink), and the laser-induced defunctionalized lines (orange) with zoom-ins on the *G* and *2D* band showing band shifts appearing after the high power laser irradiation.

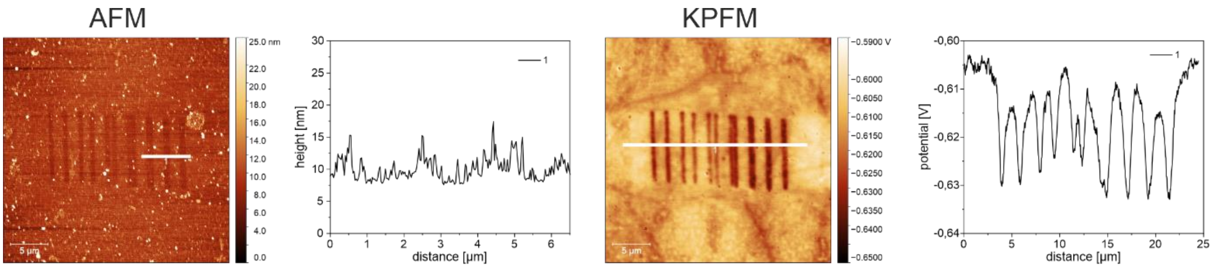

Figure SI 18: AFM and KPFM images of the laser-induced defunctionalization resolution investigation with the corresponding height and potential profiles.

3.7. Rewriting after 'Erasing'

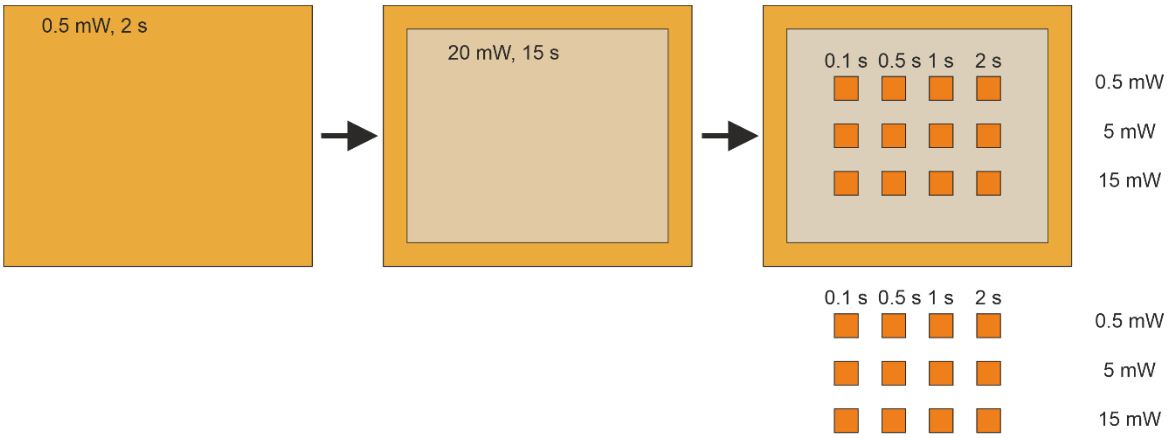

Figure SI 19: Schematic representation of the applied pattern structure to investigate the spatially resolved laser-induced defunctionalization and refunctionalization showing the initial 'writing' conditions (left) and the laser-induced defunctionalization parameters (middle), and the laser induced 'rewriting' with DBPO as well as the reference 'writing' on pristine graphene.

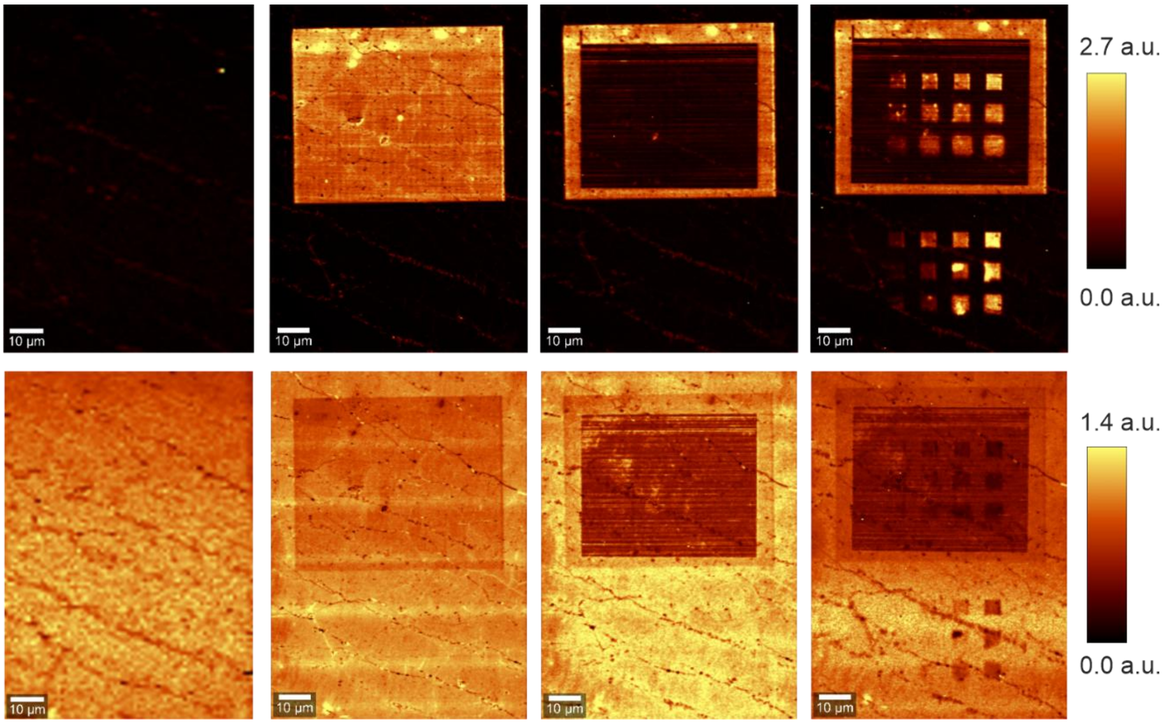

Figure SI 20: I_D/I_G (top) and I_{2D}/I_G (bottom) readout Raman mappings monitoring the laser-triggered functionalization of graphene with DBPO, the laser-induced erasing, and laser-triggered refunctionalization with DBPO from left to right.
'Writing' parameters: λ = 532 nm, P_L = 0.5 mW, t = 2 s; 65 x 55 µm, 130 x 110 points.
First 'reading' parameters: λ = 532 nm, P_L = 5 mW, t = 0.5 s; 80 x 150 µm, 240 x 450 points.
'Erasing' parameters: λ = 532 nm, P_L = 20 mW, t = 15 s, 55 x 45 µm, 110 x 90 points.
Second 'reading' parameters: λ = 532 nm, P_L = 5 mW, t = 0.5 s, 80 x 150 µm, 240 x 450 points.
'Rewriting' and reference 'writing' parameters: λ = 532 nm, P_L = 0.5 mW, 5 mW and 15 mW, t = 0.1 s to 2 s, 5 x 5 µm, 10 x 10 points.
Third 'reading' parameters: λ = 532 nm, P_L = 5 mW, t = 0.5 s, 80 x 150 µm, 240 x 450 points.

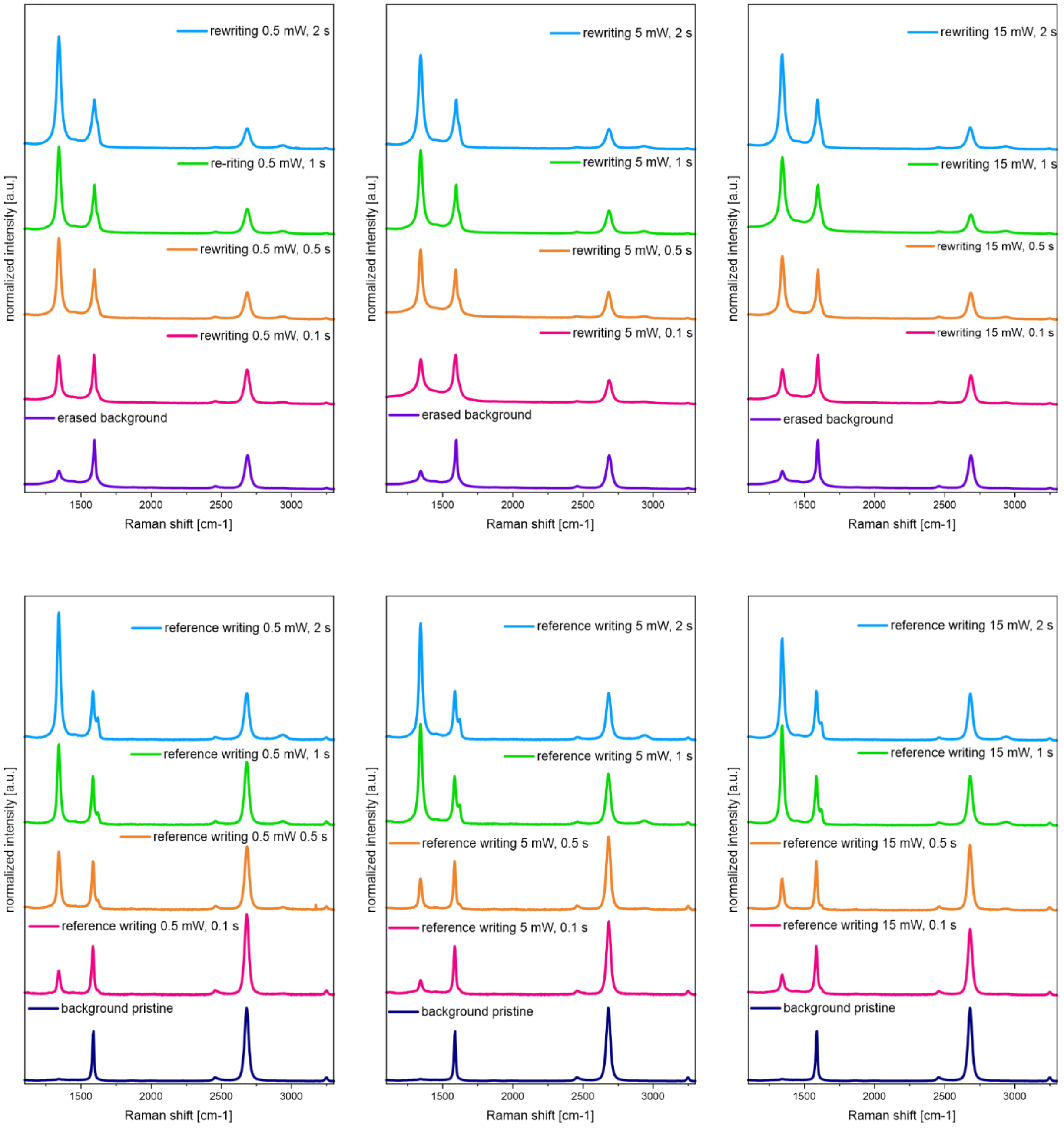

Figure SI 21: Normalized mean Raman spectra of the laser ‘writing’ on laser ‘erased’ graphene (top) and non-modified graphene (bottom).

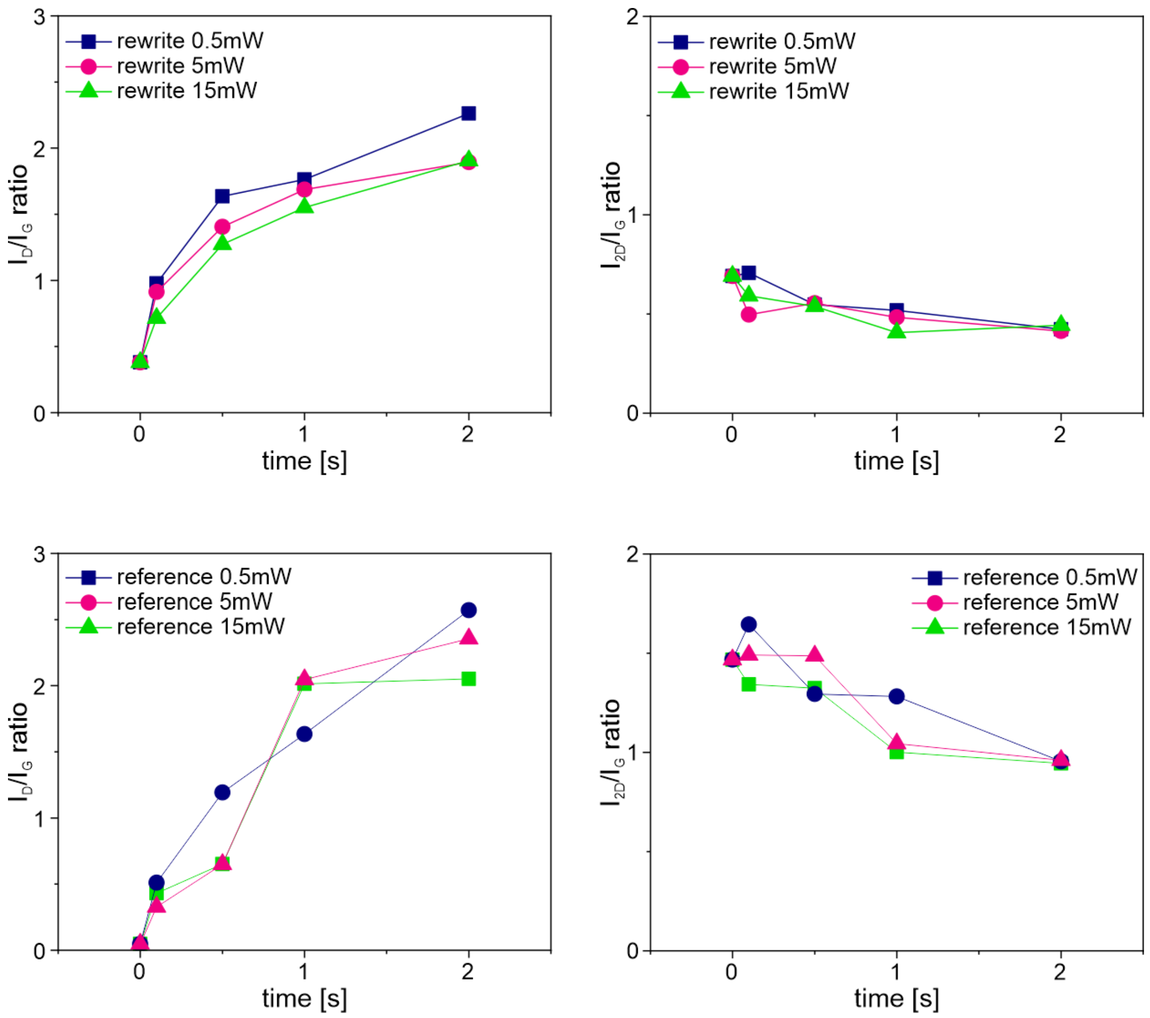

Figure SI 22: Dependency of the I_D/I_G ratio (left) and I_{2D}/I_G ratio (right) on the applied irradiation time and laser power for the laser 'writing' procedure applied using DBPO on the previously defunctionalized area (top) and a non-functionalized area as reference (bottom).

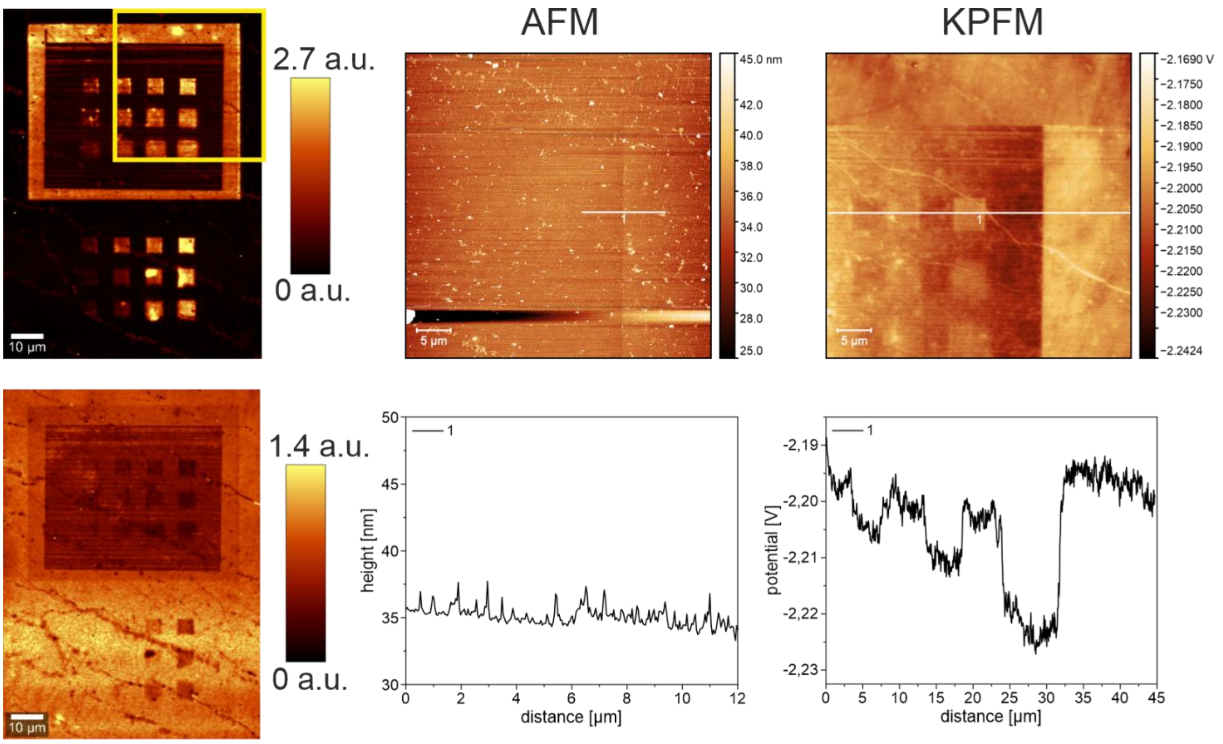

Figure SI 23: Raman maps indicating the exact area of the presented AFM and KPFM images of the refunctionalized area with the corresponding height and potential profiles.

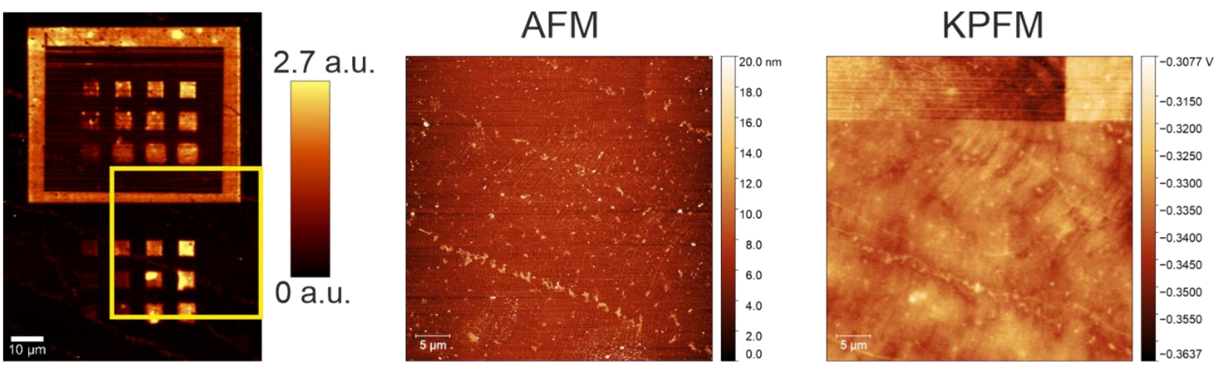

Figure SI 24: Raman map indicating the exact area of the presented AFM and KPFM images of the reference functionalized area.

3.8. Long-Term Stability

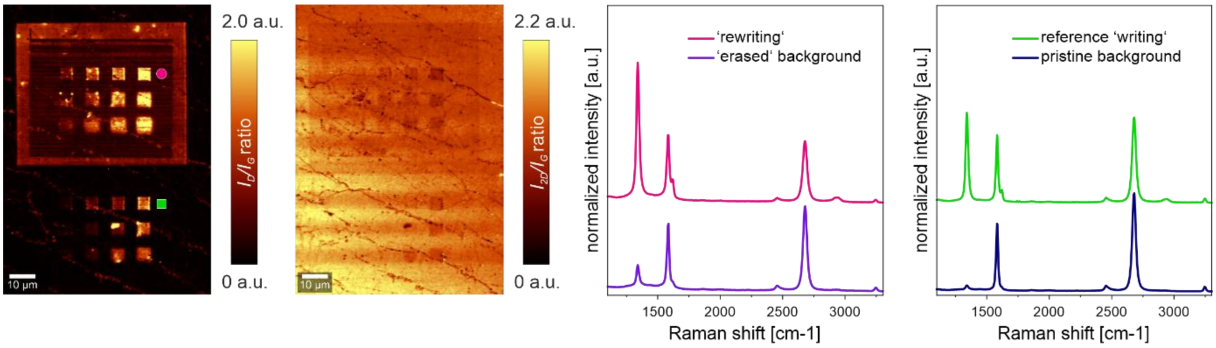

Figure SI 25: I_D/I_G 'readout' Raman mapping after the stepwise functionalization, local defunctionalization and refunctionalization of graphene with DBPO after 13 months with exemplary Raman spectra of each area.

3.9. Multiple Cycles 'Rewriting' after 'Erasing'

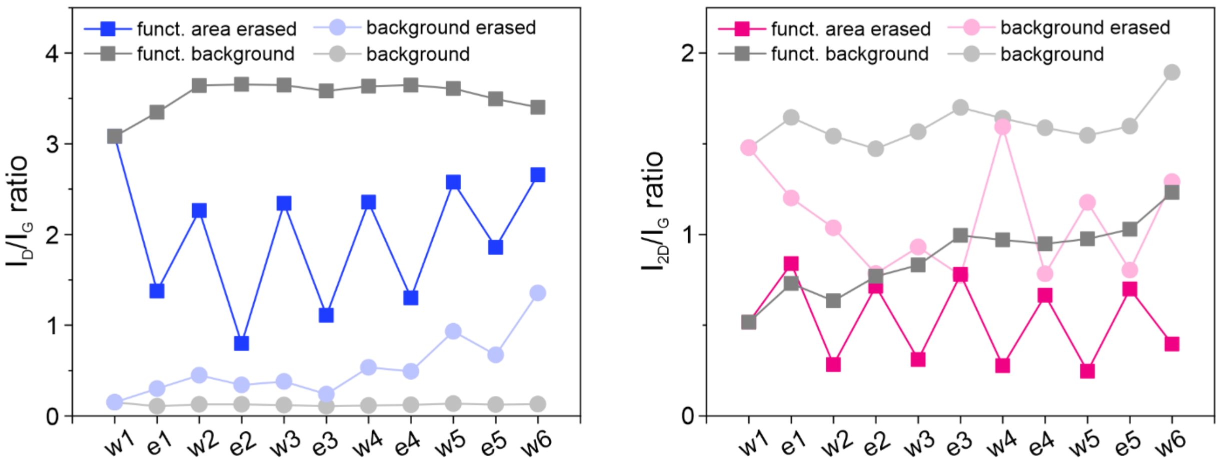

Figure SI 26: I_D/I_G (left) and I_{2D}/I_G (right)ratios for a multi-cycle procedure of 'writing' and 'erasing' covalent functionalization of graphene with DBPO in a laser based 'writing' step (w) and a laser-triggered 'erasing' step (e).

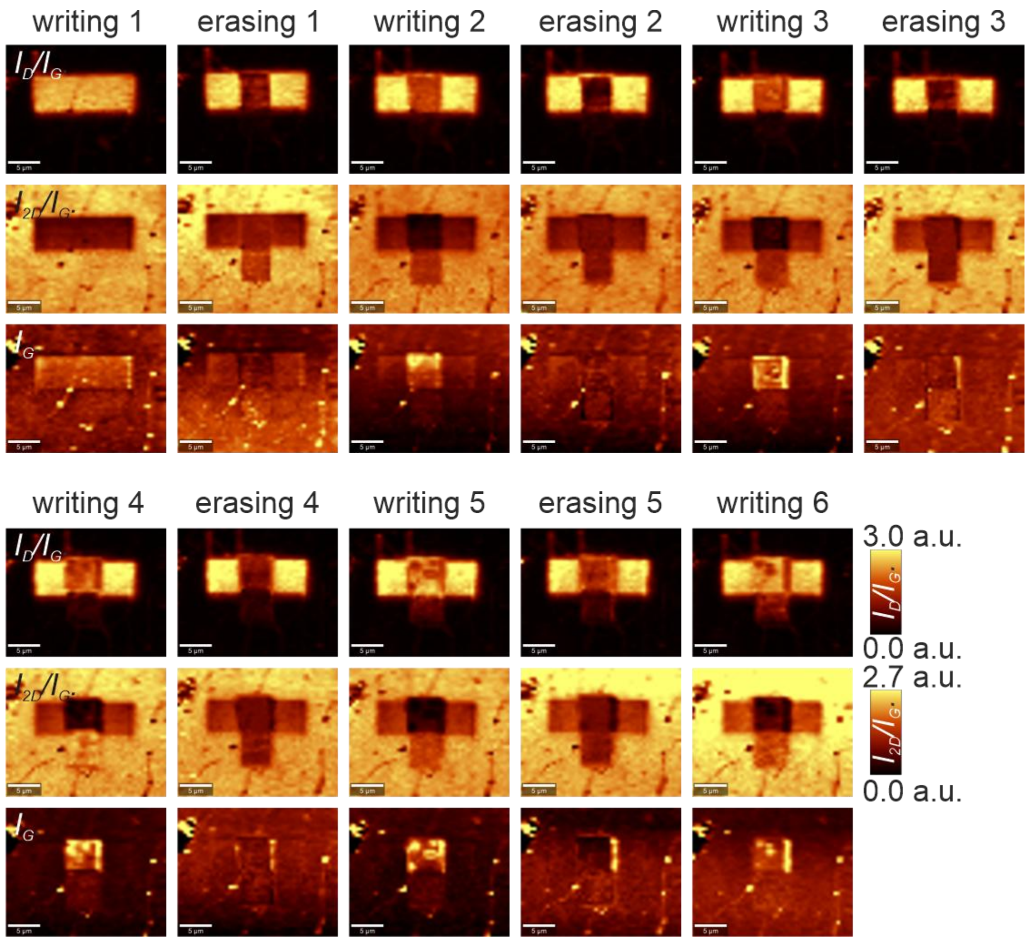

Figure SI 27: ID/IG and I2D/IG ratio as well as g band mapping after each step.

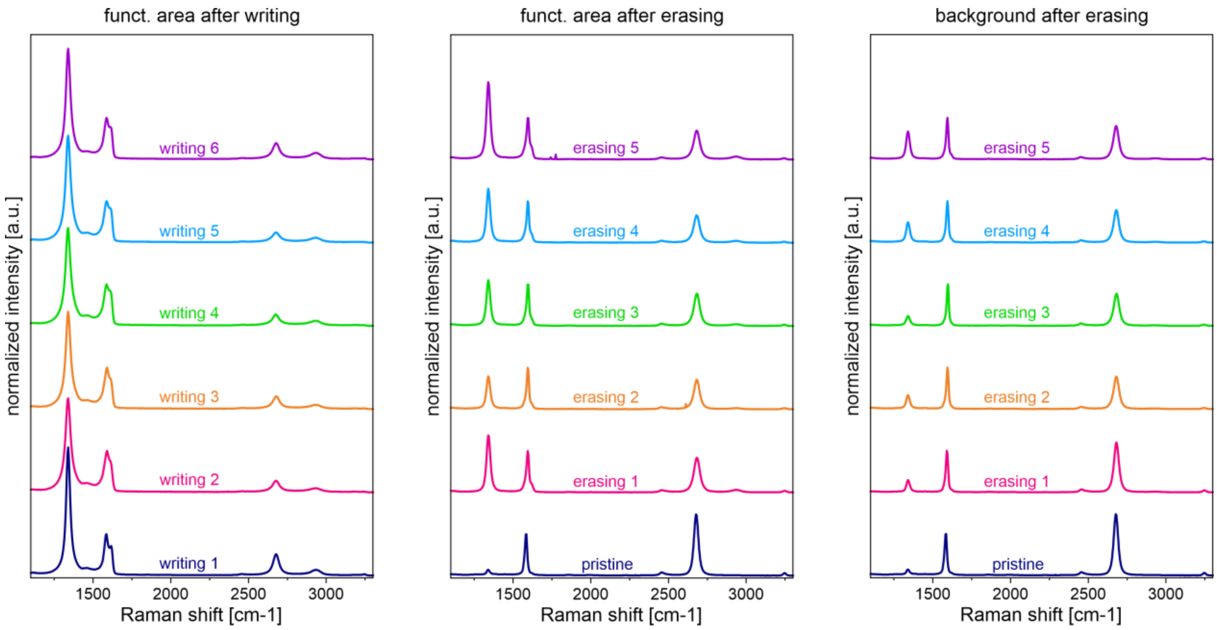

Figure SI 28: Normalized mean Raman spectra after each step.

3.10. Laser 'Erasing' on Wafer Scale Functionalized Graphene

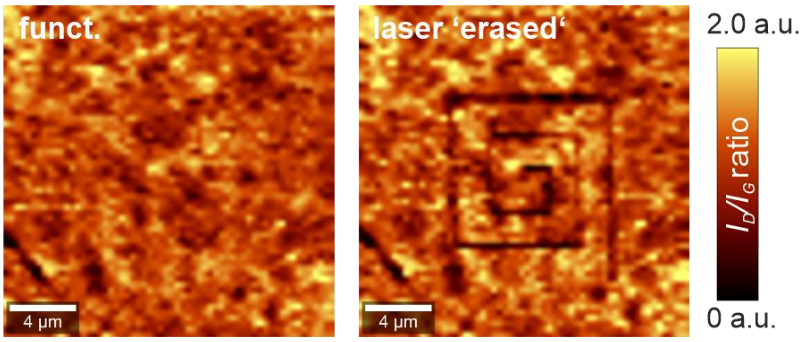

Figure SI 29: I_D/I_G Raman mappings of reductively functionalized graphene with bis(4-tert-butylphenyl) iodonium hexafluorophosphat before (left) and after (right) local defunctionalization generated by the presented laser 'erasing'.
Reductive functionalization according to Schäfer et al.[3]
First 'reading' parameters: λ = 532 nm, P_L = 5 mW, t = 0.5 s; 20 x 20 µm, 60 x 60 points.
'Erasing' parameters: λ = 532 nm, P_L = 21 mW, t = 15 s, line scans with 0.2 µm step size.
Second 'reading' parameters: λ = 532 nm, P_L = 5 mW, t = 0.5 s; 20 x 20 µm, 60 x 60 points.

References:

[1] T. Nagel, S. Wolff, S. Feng, H. Weber, J. Maultzsch, F. Hauke, A. Hirsch, Towards precision controlled 2D functional group patterning of graphene via laser writing, *Carbon* **2025**, *241*, 120376.

[2] Y. Zhang, L. Guo, S. Wei, Y. He, H. Xia, Q. Chen, H.-B. Sun, F.-S. Xiao, Direct imprinting of microcircuits on graphene oxides film by femtosecond laser reduction, *Nano Today* **2010**, *5*, 15-20.

[3] R. A. Schäfer, K. Weber, M. Koleśnik-Gray, F. Hauke, V. Krstić, B. Meyer, A. Hirsch, Substrate-Modulated Reductive Graphene Functionalization, *Angew.Chem. Int. Ed.* **2016**, *55*, 14858-14862.
